# Orthostatic blood pressure fluctuations and risk of falls in community-dwelling older adults: the role of recovery time

**DOI:** 10.1186/s12877-026-08516-1

**Published:** 2026-10-28

**Authors:** Valeria Arbieto Alvarez, Clara Hertneck, Dietrich Rothenbacher, Michael Denkinger, Dhayana Dallmeier

**Affiliations:** 1 Institute of Geriatric Research, Ulm University Medical Center, Ulm, Germany; 2 Institute of Epidemiology and Medical Biometry, University Ulm, Ulm, Germany; 3 Research Unit on Ageing, Agaplesion Bethesda Clinic Ulm, Ulm, Germany; 4 Dept. of Epidemiology, Boston University School of Public Health, Boston, MA USA

**Keywords:** Orthostatic hypotension, Orthostatic hypertension, Postural hypotension, Falls, Community-dwelling older adults

## Abstract

**Background:**

Available literature related to the association between orthostatic blood pressure (BP) variations and falls is controversial. We analyzed how orthostatic hypo- (OH) and hypertension (OHTN) relate to falls considering recovery time (RT) in community-dwelling older adults.

**Methods:**

This analysis uses data from the ActiFE cohort. BP was measured lying, immediately, 1, 2 and 3 min after standing. Systolic OH (sOH) and systolic OHTN (sOHTN) indicate a systolic BP (SBP) drop or increment ≥20 mm Hg, respectively. OH and OHTN further consider a diastolic BP (DBP) drop or increment ≥10 mm Hg. RT was categorized as Early Recovery ( < 1 min), Delayed Recovery (1-3 min), or No Recovery. Falls over 12 months were collected prospectively. Logistic regression examined associations with falls, adjusting for age, sex, diabetes, hemoglobin, instrumental activities of daily living, hand strength, fall risk-increasing drugs, past falls, Falls Efficacy Scale-International and anxiety.

**Results:**

Among 509 subjects (mean age 79 years, 44% female, 168 falls), prevalences were: 16.5% sOH, 26.9% OH, 9.0% sOHTN and 15.5% OHTN. Overall, OH was not associated with falls. However, falls risk increased with Delayed (OR 3.11 [95% CI 1.21-8.01]) and NoRecovery (OR 4.04 [95% CI 1.07-15.26]). sOH showed an adjusted OR of 2.00 [95% CI 1.15-3.47] for at least one fall. Regarding RT the risk of falls was increased in Delayed (OR 4.73 [95% CI 1.71-13.10]) and No Recovery (OR 5.06 [95% CI 1.42-22.27]). OHTN was not associated with falls.

**Conclusions:**

Overall, only sOH was associated with falls. When RT was considered, those with Delayed and No Recovery showed higher risk of falls regardless of the OH definition used.

## Highlights

- The prevalence of orthostatic blood pressure fluctuations in older adults varied considerably depending on the definition used.
- Overall, only systolic orthostatic hypotension was associated with falls, both with and without consideration of recovery time
- Models considering recovery time from orthostatic hypotension showed an increased risk for falls among those with Delayed and No Recovery, regardless of the definition used.

## Background

Falls are the leading cause of injury-related death among adults aged 65 years or older. [1] Orthostatic hypotension (OH), affecting approximately 30% of individuals aged 70 and over, has been proposed as a potential risk factor for falls in this population. [2, 3] Falls are also associated with higher mortality rates in older adults. [4] Consequently, OH and other blood pressure (BP) fluctuations have become an important focus of research aiming to improve fall prevention.

Most studies evaluating the association between OH and falls use the consensus definition, characterizing OH as a drop in systolic BP (SBP) ≥ 20 mm Hg or diastolic BP (DBP) ≥ 10 mm Hg within the first three minutes after standing. [5] However, few studies assess OH based solely on systolic values [3, 6] or consider the BP recovery time pattern after standing. As a result, the role of recovery time (RT) from orthostatic BP changes in relation to falls remains unclear. Furthermore, most studies focus mainly on the first minute after standing [7, 8], emphasizing the need for further research covering the full three-minute period [9, 10]. In addition, patient stratification according to BP recovery patterns relative to baseline values has rarely been explored.

Orthostatic hypertension (OHTN), defined as a rise in SBP ≥ 20 mm Hg or DBP ≥ 10 mm Hg within three minutes after standing, occurs in approximately 1–28% of adults and becomes more prevalent with age. [11] Previous studies have linked OHTN to increased mortality and cardiovascular disease (CVD) risk in older adults [12, 13]. However, research investigating OHTN, RT, and falls remains limited. Therefore, this study aimed to analyze the following two entities for orthostatic BP fluctuations: OH and OHTN, and their association with falls in community-dwelling older adults, while evaluating RT during the first three minutes after standing as a potential effect modifier.

## Methods

### Study population

We derived the study population from the ActiFE study (Activity and Function in the Elderly in Ulm), designed as a prospective, observational population-based cohort study initiated in 2009, including the community-dwelling population of older adults in Ulm and near regions (n = 1506). Inclusion criteria were age 65 - 90 years and the ability to walk through their own room with or without devices, indicating independent indoor ambulation (participation at baseline was 20%). Individuals with severe deficits in cognition, vision, and hearing, or with impaired German language comprehension were excluded [14]. The present analyses used data from the seven-year follow-up examination performed between 2016 and 2017 (n = 526). All assessments were completed within one week, comprising two visits either to the study center or, for participants unable to attend in person, conducted at their home. All participants gave written consent. The study protocol was approved by the ethical committee of Ulm University (application no. 318/08 and 50/12). The detailed version of the ActiFE study protocol is available [14].

Out of 526 participants at the seven-year examination, we excluded a total of 17 patients, leading to n = 509. Information on falls was missing for 12 participants. An additional five participants experienced a concomitant rise in SBP of ≥20 mmHg and a drop in DBP of ≥10 mmHg after standing, preventing a classification according to the defined criteria for orthostatic fluctuations. These participants were therefore excluded from the analysis. Furthermore, for multivariable statistical analyses, we excluded up to 59 observations due to missing data on the considered covariables.

**Fig. 1:**
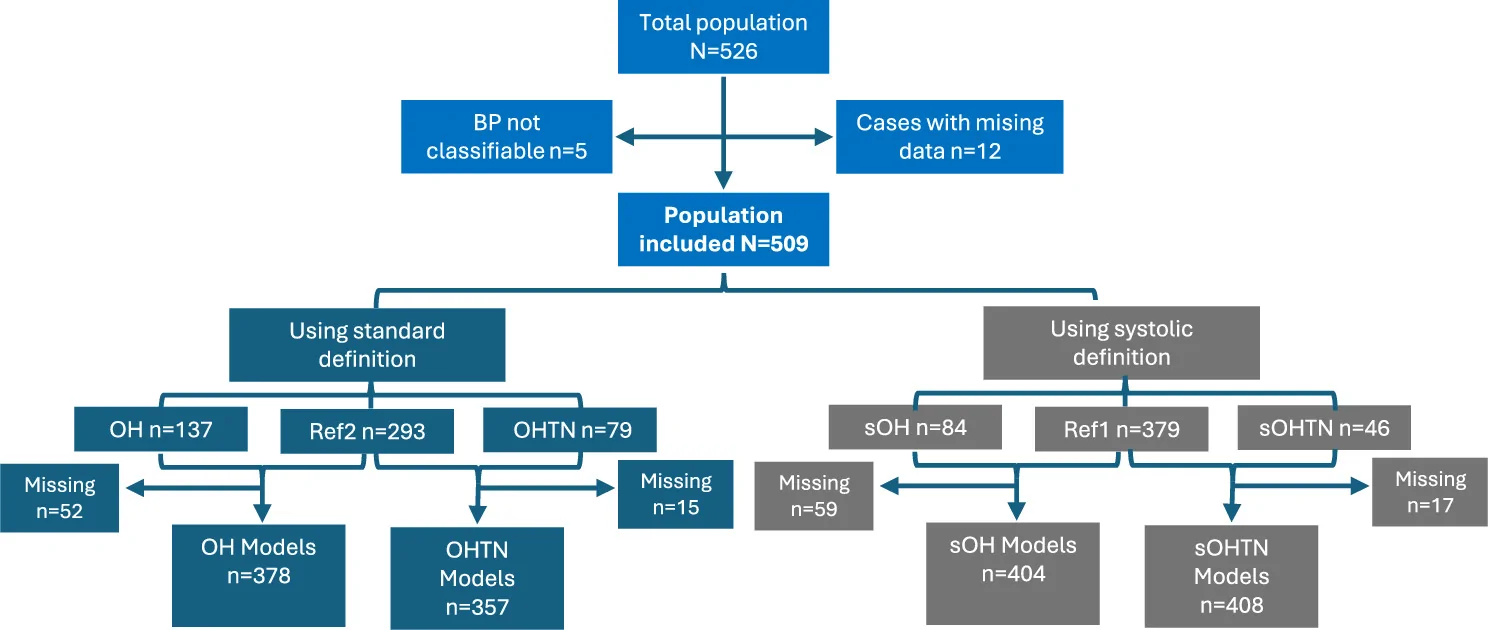
Overview of Populations

OH and OHTN represent two distinct clinical entities with different underlying pathophysiological mechanisms and clinical implications. Crucially, to ensure a clean and rigorous comparison, we designed our reference group to consist strictly of individuals without any blood pressure fluctuations upon orthostatic stress. Figure 1(Overview of populations) shows the study populations considered for the four analyses: For OH we excluded 79 OHTN cases and 52 cases due to missing data, leading to 378 participants. For systolic OH (sOH) we excluded 46 systolic OHTN (sOHTN) cases and 59 cases due to missing in the covariates, remaining 404 participants. OHTN analysis included 357 participants, due to exclusion of 137 OH cases and 15 cases with missing data. For sOHTN 84 OH cases and 17 cases due to missing in the covariates were excluded, leading to 408 participants. We defined a reference population for each analysis, Reference 1 (Ref 1) and Reference 2 (Ref 2), consisting of participants without orthostatic hypo- and hypertensive BP changes, respectively. Ref 1 (n = 379) included participants who experienced neither a rise nor a drop in SBP and did not meet the criteria for sOH or sOHTN. Ref 2 (n = 293) comprised participants without OH or OHTN.

### Data collection

We obtained demographic data through standardized questionnaires. Age was modeled as a linear continuous variable, and the assumption of linearity in the logit was formally evaluated and confirmed using the Box-Tidwell test. Medical comorbidities used the items listed in the Functional Comorbidity Index [15]. Further clinical information such as height, weight and heart rate was digitally measured. Questionnaires included activities of daily living (ADL), instrumental activities of daily living (IADL) and the Hospital Anxiety and Depression Scale (HADS). We assessed fear of falling using the short version of the Falls Efficacy Scale-International (s-FES-I). Participants were interviewed about any previous falls within the past 12 months. We assessed physical performance using the Short Physical Performance Battery (SPPB) test and measured handgrip strength with a JAMAR dynamometer (Sammons Preston, Bolingbrook, Il (USA)). We determined hemoglobin levels (g/dl) through blood samples with separate consent. Information on medication was collected by scanning barcodes of the medications at the participant´s home or the ones brought during the visit. Changes in medication were documented using a medication calendar. We categorized Fall Risk Increasing Drugs (FRIDs) according to the Screening Tool of Older Persons Prescriptions (STOPPFALL) [16]. The 7-year follow-up (between 2016 and 2017) comprises two visits at the beginning and end of the one-week examination period. Orthostatic BP measurements were performed at visit one. A fall calendar was provided at the same visit one. The fall events analyzed in this paper occurred and were recorded prospectively over the following 12 months.

### Blood pressure fluctuations and recovery groups

We assessed orthostatic changes in BP using the active standing test. For this purpose, we instructed participants to lie down for ten minutes, during which BP was measured every two minutes. The last BP measurement during the 10-minute period was defined as the supine baseline BP measurement. After ten minutes participants were asked to stand up, where BP and heart rate were measured immediately. We obtained following BP measurements immediately and at 1, 2 and 3 min after standing. Any symptom of dizziness, nausea or blurry vision after standing up was documented. BP measurements were performed using an A&D Medical UA-767BT Digital BP Monitor. The 1,2-, and 3-minute time points were referred to as the timeframe where the measurement started.

For this analysis, OH and OHTN are defined through the measurement obtained immediately after standing compared to the supine BP. The BP drop qualified as OH if it adhered to the consensus criteria of a SBP drop ≥20 mm Hg and/or a DBP drop ≥10 mm Hg [17]. sOH refers to those cases where only a SBP drop ≥20 mm Hg applied. OHTN was characterized by an SBP increment ≥20 mm Hg and/or DBP ≥ 10 mm Hg according to the consensus criteria [11]. Lastly, sOHTN was created in analogy for sOH, considering only an SBP increment ≥20 mm Hg. Subsequent BP measurements obtained at 1,2, and 3 min after standing were used to characterize the temporal recovery pattern following initial orthostatic BP change. These measurements were used to define RT categories. The BP fluctuations were coded as categorical variables: binary for the primary analyses, and as a 4-level variable by consideration of the RT.

**Fig. 2:**
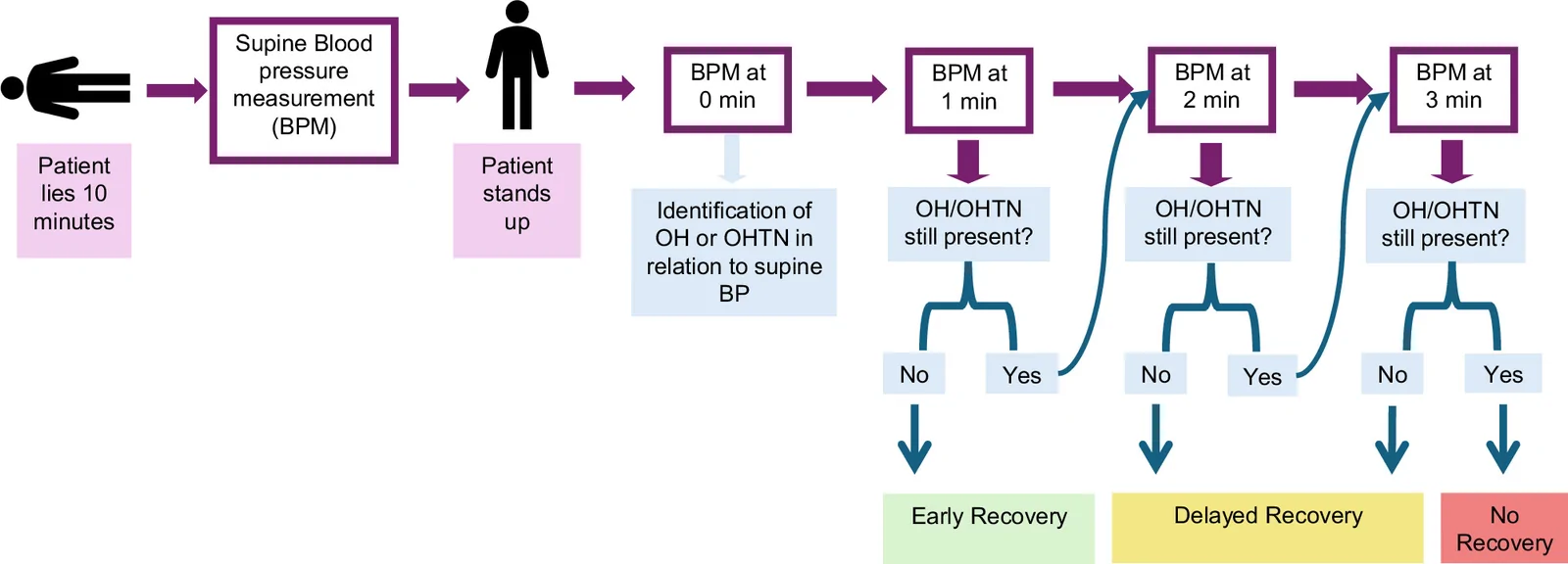
Identification of BP fluctuations and classification of recovery groups

We defined RT from a BP fluctuation as the earliest point in time at which BP no longer met orthostatic criteria. RT groups were formed for each of the four definitions, and further stratified based on the temporal pattern of the BP response during the three-minute standing phase: “Early Recovery” defined as a BP fluctuation that resolved by the one-minute measurement, “Delayed Recovery” referred to a fluctuation, which persisted at the first minute but resolved by the second or third minute, “No Recovery” defined a sustained presence of OH or OHTN through the entire three-minute standing period (Fig. 2**- **Identification of BP fluctuations and classification of recovery groups).

### Falls

We assessed falls prospectively over a one-year follow-up after the baseline examinations. Falls were recorded using a falls calendar, which was handed out at the first visit during the seven-year examination and filled out by the participants on a weekly basis as recommended by the Prevention of Falls Network Europe [18]. The study center collected information from the calendars every 3 months to minimize information bias. In order to maximize participants’ compliance, reminders were sent out every 6 weeks. If the calendars were not returned within 2 weeks, we contacted participants by phone. A fall was defined as an event which results in a person coming to rest inadvertently on the ground or floor or other lower level [19]. The prospectively recorded falls were the primary outcome of this study. Participants with at least one fall were considered as “fallers”.

### Statistical analysis

We classified participants into fallers or non-fallers according to the reported falls during the 12-month follow-up period. A descriptive analysis examined the demographic characteristics of the population, according to falls and BP fluctuation. We present categorical variables as counts and percentages. Normally distributed continuous variables are shown with their mean and standard deviation, while non-normally distributed continuous variables are shown with their median and interquartile ranges. For comparisons between categorical variables, we used the Chi-Square test and Fisher Exact test. The T-test was used for normally distributed continuous variables and the Mann-Whitney-U test for non-normally distributed variables.

We analyzed the association between each BP fluctuation and falls with binary logistic regression models, followed by an analysis where each BP fluctuation was categorized considering the RT (Early, Delayed, and No Recovery). Covariates were selected based on a priori literature [20–25] consensus regarding blood pressure fluctuations and fall risk. The covariates selection was supplemented as needed in cases population-specific confounding was identified. A consistent core model adjusting for age, sex, diabetes, prior falls, IADL score, and psychological metrics (HADS-Anxiety, s-FES-I) was applied to both conditions. The OH models were further adjusted for handgrip strength and FRIDs, while the OHTN models were specifically adjusted for baseline hemoglobin levels and diuretic medication use, both of which demonstrated strong significance in the OHTN baseline analysis. With respect to the adjustment for baseline SBP and DBP, baseline DBP was not associated with either the outcome or blood pressure fluctuations. Therefore, it was not considered a confounder. Baseline SBP was not associated with falls. However, it was statistically significantly associated with sOH and sOHTN (see Additional Files 1–4). We then performed a secondary analysis by adding baseline SBP to the adjusted model to explore its effect on the association. Overall, few missings were detected for the identified confounders, representing less than 1.6% of the data entries (Additional Files 9-12). For all statistical models a two-sided significance level of α = 0.05 was used. The estimated OR are provided with the 95^th^ percent confidence intervals (CI). We conducted all analyses using IBM SPSS Version 28.

## Results

In the study population (N = 509, median age 79 years, 44.0% female), we identified 168 (33.0%) participants as fallers, with 100 (59.5%) reporting one fall, and 68 (40.5%) multiple falls during the 12 months follow-up period. Overall, fallers reported more frequent previous falls in the past 12 months and had higher median scores in ADL, IADL, and s-FESI. Furthermore, fallers had a higher prevalence of arthritis, arthrosis, peripheral arterial disease, a higher median number of drugs prescribed and a greater proportion taking four or more FRIDs, including diuretics compared to non-fallers (Table 1).

**Table 1:** Participant characteristics

	Instrument range	Total N = 509	Fallers n = 168, (33%)	Non-fallers n = 341, (67%)
Falls, n (%)	1		100 (59.5)	
	≥2		68 (40.5)	
Age (y), median (Q1, Q3)		79 (76, 82)	79 (76, 82)	79 (76, 83)
Sex (female), n (%)		224 (44)	81 (48.2)	143 (41.9)
Gait speed (m/s), median (IQR)		1.2 (0.3)	1.2 (0.3)	1.2 (0.3)
Gait speed <0.8 m/s, n (%)		45 (8.8)	20 (11.9)	25 (7.3)
Hand grip strength (kg), mean (SD)				
Female		19.23 (6.6)	18.2 (6.3)	19.8 (6.7)
Male		33.13 (8.5)	32.66 (8.2)	33.3 (8.6)
**Falls in the prior 12 months, n (%)**		**142 (28.1)**	**69 (41.8)**	**73 (21.5)**
**ADL**^**a**^**score, median (IQR)**	**0-6**	**0 (2)**	**1 (3)**	**0 (1)**
**FI**^**b**^**Rockwood, median (IQR)**	**0-1**	**0.2 (0.1)**	**0.2 (0.1)**	**0.2 (0.1)**
**Frail with FI**^**b**^ ≥ **0.2/ Frailty (yes/no), n (%)**		**123 (31.8)**	**53 (41.7)**	**70 (26.9)**
**iADL**^**c**^**score, median (IQR)**	**0-8**	**1 (3)**	**2 (4)**	**1 (2)**
HADS^d^ Score depression, median (IQR)	0-21	4 (4)	4 (4)	4 (4)
HADS Score anxiety, median (IQR)	0-21	4 (4)	4 (4)	4 (4)
**Short FES-I**^**e**^**score, median (IQR)**	7-28	**8 (3)**	**9 (2)**	**8 (3)**
**Comorbidities, n (%)**				
**Arthritis**		39 (7.8)	21 (12.7)	18 (5.3)
**Arthrosis**		227 (45.0)	91 (54.8)	136 (40.2)
Heart failure		78 (15.6)	33 (19.9)	45 (13.4)
Arterial Hypertension		299 (59.0)	96 (57.1)	203 (59.9)
**Peripheral arterial disease**		94 (18.7)	40 (24.1)	54 (16.0)
Diabetes		66 (13)	28 (16.7)	38 (11.2)
Depression		44 (8.7)	20 (12.0)	24 (7.1)
**Hemoglobin (g/dL), median (IQR)**		**13.7 (17)**	**13.5 (15)**	**13.8 (17)**
**Number of drugs, median (IQR)**		**4 (4)**	**5 (5)**	**4 (4)**
**Use of diuretics, n (%)**		**195 (38.3)**	**75 (44.6)**	**120 (35.2)**
Use of antihypertensives, n (%)		362 (71.1)	118 (70.2)	244 (71.6)
FRIDs^f^ intake, n (%)		377 (81.6)	119 (76.8)	258 (84.0)
**Number of FRIDs, n (%)**				
0		**85 (18.4)**	**36 (23.2)**	**49 (16.0)**
1		**125 (27.1)**	**35 (22.6)**	**90 (29.3)**
2		**125 (27.1)**	**29 (18.7)**	**96 (31.3)**
3		**85 (18.4)**	**36 (23.2)**	**49 (16.0)**
≥4		**42 (9.1)**	**19 (12.3)**	**23 (7.6)**
Orthostatic Hypotension, n (%)				
OH^g^		137 (26.9)	45 (26.8)	92 (27.0)
**sOH**^**h**^		**84 (16.5)**	**38 (22.6)**	**46 (13.5)**
Orthostatic Hypertension, n (%)				
OHTN^i^		79 (15.5)	22 (13.1)	57 (16.7)
sOHTN^j^		46 (9.0)	11 (6.5)	35 (10.3)
Baseline SBP (at 10 min of lying down) (mm Hg), median (Q1, Q3)		128 (118, 140)	128 (117, 143)	128 (118, 139)
Baseline DBP (at 10 min of lying down) (mm Hg), median (Q1, Q3)		73 (69, 78)	73 (69, 79)	74 (69, 79)

^a^*ADL* activities of daily living, ^b^*FI* frailty index, ^c^*iADL* instrumental activities of daily living, ^d^*HADS* hospital anxiety and depression scale, ^e^*FES-I* falls efficacy scale- international, ^f^*FRIDs* fall risk inducing drugs, ^g^*OH* orthostatic hypotension, ^h^*sOH* systolic OH, ^i^*OHTN* orthostatic hypertension, ^j^*sOHTN* systolic OHTN

Due to missings as described in methods the n considered varies for some variables.

There were 137 (26.9%) participants with OH, and 84 (16.5%) with sOH. The OHTN group included 79 (15.5%) participants while sOHTN was found in 46 (9.0%) of them. The reference populations included 293 (57.6%) (Ref 1) and 379 (74.4%) (Ref 2) individuals for the OH/OHTN and for sOH/sOHTN analyses, respectively (Fig. 1).

### Orthostatic hypotension and falls

Overall, 146 participants (34.0%) reported at least one fall, with no significant difference based on OH status. Among the 137 participants with OH, the majority demonstrated Early Recovery (n = 103, 75.2%), followed by Delayed Recovery (n = 24, 17.5%) and No Recovery (n = 10, 7.3%). Notably, the proportion of fallers increased as blood pressure recovery took longer, affecting 25.2% of the Early Recovery group, 54.2% of the Delayed Recovery group, and 60.0% of those with No Recovery. OH individuals had a lower percentage of women, on average faster gait speed, lower s-FES-I and HADS Anxiety scale scores, using more FRIDs, particularly torasemide, doxazosin and tamsulosin compared to the reference group. Diabetes prevalence was nearly twice as high in the OH group (Additional File 1).

The relationship between OH and falls was not statistically significant overall. However, multivariable analysis showed a statistically significant association when considering the RT, with an increased falls risk for those with a Delayed Recovery (OR: 3.11, 95% CI [1.21, 8.01]) and with No Recovery (OR: 4.04, 95% CI [1.07, 15.26]), when compared to those without OH. A further adjustment for baseline SBP as in our secondary analysis showed a strengthening of this association among those with No Recovery (OR: 4.43, 95% CI [1.13, 17.30] (Table 2, Additional File 5).

**Table 2:** Logistic regression between OH^1^ and falls (n = 378, 128 falls)

	Participants with falls/n	Model^a^	Model^b^	Recovery Categories	Participants with falls/n	Model^a^	Model^b^
No OH	89/255	Ref.	Ref.	No OH	89/255	Ref.	Ref.
OH	39/123	0.96 [0.58, 1.58]	0.94 [0.57, 1.56]	OH with Early Recovery	20/91	0.57 [0.31, 1.03]	0.56 [0.30, 1.01]
				OH with Delayed Recovery	13/22	**3.11 [1.21, 8.01]**	**3.19 [1.23, 8.30]**
				OH with No Recovery	6/10	**4.04 [1.07, 15.26]**	**4.43 [1.13, 17.30]**

^a^Adjusted for age, sex, diabetes, instrumental Activities of Daily Living (iADL) score, hand grip strength, Fall Risk-Increasing Drugs (FRIDs), falls in the prior 12 months, Falls Efficacy Scale-International (FES-I) score, Hospital Anxiety and Depression Scale (HADS)-anxiety score.

^b^Adjusted for age, sex, diabetes, instrumental Activities of Daily Living (iADL) score, hand grip strength, Fall Risk-Increasing Drugs (FRIDs), falls in the prior 12 months, Falls Efficacy Scale-International (FES-I) score, Hospital Anxiety and Depression Scale (HADS)-anxiety score, SBP baseline.

^1^OH - Orthostatic Hypotension

Odds ratios (ORs) and 95% confidence intervals (CIs) for the association between OH and falls

### Systolic orthostatic hypotension

We observed a statistically significant difference for falls between those with sOH versus the reference population (45.2% versus 18.1%). When it came to the recovery, 56 (66.7%) showed Early, 20 (23.8%) Delayed and eight (9.5%) No Recovery. In analogy to OH, the incidence proportion of fallers was higher the longer the RT (37.5%, 60.0% and 62.5%, respectively). Individuals with sOH showed lower HADS anxiety scale score, with higher prevalence for diabetes (23.8% versus 11.4%) and on average lower DBP (74.1 vs. 75.0 mm Hg). Participants with sOH were using more FRIDs, particularly torasemide, and tamsulosin, as well as anticoagulants and statins when compared to the reference population (Additional File 2).

Multivariable regression analysis showed a higher falls risk among those with sOH compared to the reference population with an OR of 2.00 (95% CI 1.15, 3.47). There was a significantly increased fall risk among those with Delayed Recovery with an OR of 4.73 (95% CI 1.71, 13.10) and those with No Recovery with an OR 5.06 (95% CI 1.15, 22.27) when compared to the reference population. A further adjustment for baseline SBP as in our secondary analysis showed a strengthening of the association among those with No Recovery (OR: 5.97, 95% CI [1.30, 27.38] (Table 3).

**Table 3:** Logistic regression between sOH^1^ and falls, (n = 404, 137 falls)

	Participants with falls/n	Model^a^	Model^b^	Recovery categories	Participants with falls/n	Model^a^	Model^b^
No sOH	104/329	Ref.	Ref.	No sOH	104/329	Ref.	Ref.
sOH	33/75	2.00 [1.15, 3.47]	**2.05 [1.17, 3.61]**	sOH with Early Recovery	16/48	1.20 [0.60, 2.38]	1.23 [0.62, 2.45]
				sOH with Delayed Recovery	12/19	**4.73 [1.71, 13.10]**	**4.97 [1.78, 13.87]**
				sOH with No Recovery	5/8	**5.06 [1.42, 22.27]**	**5.97 [1.30, 27.38]**

^a^Adjusted for age, sex, diabetes, instrumental Activities of Daily Living (iADL) score, hand grip strength, Fall Risk-Increasing Drugs (FRIDs), falls in the prior 12 months, Falls Efficacy Scale-International (FES-I) score, Hospital Anxiety and Depression Scale (HADS)-anxiety score.

^b^Adjusted for age, sex, diabetes, instrumental Activities of Daily Living (iADL) score, hand grip strength, Fall Risk-Increasing Drugs (FRIDs), falls in the prior 12 months, Falls Efficacy Scale-International (FES-I) score, Hospital Anxiety and Depression Scale (HADS)-anxiety score, SBP baseline.

^1^OH - Orthostatic Hypotension

Odds ratios (ORs) and 95% confidence intervals (CIs) for the association between OH and falls

### Orthostatic hypertension

Among those with OHTN, 22 (29.1%) reported at least one fall, with no statistically significant difference with respect to the reference group. The Early, Delayed and No Recovery groups consisted of 29 (36.7%), 18 (22.8%) and 32 (40.5%) participants, respectively. No association between the incidence proportion of falls and the RT was observed (Table 3). Those with OHTN had lower IADL and ADL scores when compared to the reference population. Interestingly, circulatory disorders such as history of angina pectoris were less prevalent in the OHTN group when compared to the reference. While SBP was elevated in the OHTN group (143 mm Hg versus 135 mm Hg), no major difference in the DBP was noted between the two groups. Patients with OHTN reported less often the use of diuretics (20.3% versus 38.2%), with no other differences regarding the use of FRIDs (Additional File 3). Overall, OHTN was not found to be significantly associated with falls (Table 4).

**Table 4:** Logistic regression between OHTN^1^ and falls (n = 357, 115 falls)

	Participants with falls/n	Model^a^	Model^b^	Recovery categories	Participants with falls/n	Model^a^	Model^b^
No OHTN	93/279	Ref.	Ref.	No OHTN	93/279	Ref.	Ref.
OHTN	22/78	1.05 [0.58, 1.92]	1.09 [0.59, 1.99]	OHTN with Early Recovery	6/29	0.62 [0.23, 1.67]	0.64 [0.24, 1.75]
				OHTN with Delayed Recovery	9/18	2.49 [0.88, 7.10]	2.65 [0.93, 7.59]
				OHTN with No Recovery	7/31	0.95 [0.38, 2.39]	0.95 [0.37, 2.41]

^a^Adjusted for age, sex, diabetes, instrumental Acitvities of Daily Living (iADL), falls in the prior 12 months, hemoglobin, use of diuretics, Falls Efficacy Scale-International (FES-I) and Hospital Anxiety and Depression Scale (HADS)-anxiety-score

^b^Adjusted for age, sex, diabetes, instrumental Acitvities of Daily Living (iADL), falls in the prior 12 months, hemoglobin, use of diuretics, Falls Efficacy Scale-International (FES-I), Hospital Anxiety and Depression Scale (HADS)-anxiety-score and SBP Baseline

^1^OHTN- Orthostatic Hypertension

Odds ratios (ORs) and 95% confidence intervals (CIs) for the association between OHTN and falls.

### Systolic orthostatic hypertension

Overall, only 46 participants were noted to have sOHTN, with 11 (23.9%) reporting at least one fall. We did not find a significant association between sOHTN and falls, even after consideration of the RT (Table 5). Participants with sOHTN used more frequently diuretics compared to the reference group. Otherwise, no other significant differences could be detected (Additional File 4).

**Table 5:** Logistic regression between sOHTN^1^ and falls (n = 408, 111 falls)

	Participants with falls/n	Model^a^	Model^b^	Recovery categories	Participants with falls/n	Model^a^	Model^b^
No sOHTN	111/363	Ref.	Ref.	No sOHTN	111/363	Ref.	Ref.
sOHTN	11/45	1.02 [0.48, 2.16]	1.00 [0.47, 2.16]	sOHTN with Early Recovery	3/18	0.66 [0.18, 2.44]	0.64 [0.17, 2.39]
				sOHTN with Delayed Recovery	5/14	1.51 [0.46, 5.03]	1.55 [0.47, 5.16]
				sOHTN with No Recovery	3/13	1.05 [0.27, 4.05]	1.03 [0.27, 4.00]

^a^Adjusted for age, sex, diabetes, instrumental Acitvities of Daily Living (iADL), falls in the previous 12 months, hemoglobin, use of diuretics, Falls Efficacy Scale-International (FES-I) and Hospital Anxiety and Depression Scale (HADS)-anxiety-score

^b^Adjusted for age, sex, diabetes, instrumental Acitvities of Daily Living (iADL), falls in the previous 12 months, hemoglobin, use of diuretics, Falls Efficacy Scale-International (FES-I), Hospital Anxiety and Depression Scale (HADS)-anxiety-score and SBP Baseline

^1^OHTN- Orthostatic Hypertension

Odds ratios (ORs) and 95% confidence intervals (CIs) for the association between OHTN and falls.

### Recovery groups

Descriptive analyses of baseline clinical covariates across the blood pressure fluctuation recovery groups revealed the following: For both the OH and sOH populations, a higher burden of diabetes and FRIDs was consistently tied to the Delayed and No Recovery group. Specifically, the prevalence of diabetes peaked at 33.3% for OH and 25.0% for sOH when compared to their respective reference cohorts (11.4% for both). Similarly, the median FRIDs burden was significantly higher in the Delayed and No Recovery groups for both conditions. Baseline SBP was also significantly higher in the No Recovery group than in all other groups, averaging 142 mm Hg for OH and 148.5 mm Hg for sOH. However, diastolic baseline differences were not statistically significant. (Additional File 13, Additional File 14**)**. Among those with OHTN a lower proportion of participants using diuretic medication was observed in the No Recovery group compared to the other groups. However, these results should be interpreted with caution due to the small sample size. Additionally, baseline DBP was noted to be markedly lower among those with No Recovery compared to the other groups (Additional File 15).

## Discussion

In our analysis we found that only OH defined through the systolic criteria was significantly associated with falls. After taking RT into account, those with Delayed and No Recovery had a statistically significant higher falls risk independently of the OH definition used even after adjustment for baseline SBP, highlighting the importance of considering RT when evaluating orthostatic BP fluctuations in relationship to falls. Overall, OHTN was not linked with falls.

The observed OH prevalence of 26.9% is consistent with findings of previous studies [17]. Although isolated systolic hypertension is a common condition among older adults, sOH has been seldom analyzed, with a reported prevalence between 4 and 25.3% [3, 6, 9, 26, 27]. Our 16.5% prevalence for sOH fits within this spectrum. However, results across studies are difficult to compare as the measurement methods differed, with some using the sitting to standing method, where the difference in BP is not as strong as with the lying-to-standing method [28, 29]. The head-up tilt test is the method with the highest sensitivity for detecting orthostatic BP fluctuations but is rarely used in a clinical setting as it requires more specialized equipment. Therefore the lying-to standing method remains as the recommended initial diagnostic approach. [30, 31] Lastly, most studies also set different time points during measurement. For instance our time points for measuring sOH immediately after standing capturing the largest drops in SBP are usually not reported in other literature [32].

OH has shown mixed results across literature when assessing its link to falls. A meta-analysis of previous studies found an independent link between OH and falls [2]. In our study, the association with OH and falls becomes statistically significant only when using sOH. Although some results vary in intensity, the link of sOH with falls has been congruent with the results of previous researchers [2, 28, 33, 34]. Heitterachi et al. portrayed a similar finding, where they highlight SBP as the main factor associated with this outcome, especially if the SBP had not recovered until after the third minute [35].

A similar result was observed by Gangavati et al. in which they did not detect a significant link between OH and falls, whereas they found a higher fall risk in participants with sOH after one minute of standing [6]. Furthermore, other studies have found that the standard OH definition may not be predictive of subsequent falls [36, 37]. This raises the question as to whether the systolic OH definition may be a stronger predictor of falls than the standard definition, as suggested [36, 37]. Romero-Ortuno et al. have also stated that SBP values may be more relevant than the DBP ones in the context of orthostatic intolerance [11]. Furthermore, isolated diastolic OH has proven to be rare and DBP has been shown to remain stable or increase slightly during orthostatism, whereas SBP is more prone to fall. Some researchers proposed the exclusion of DBP in the formal OH definition altogether [38].

One possible explanation for the stronger relevance of sOH in fall risk is that DBP could play a relatively smaller role in influencing stroke volume (SV) and cardiac output (CO) during orthostatism. [39] Under orthostatic stress, maintaining or increasing SV and CO is critical to sustain cerebral perfusion pressure and prevent syncope or falls. Therefore, a decline in SBP may more strongly reflect impaired hemodynamic compensation and reduced cerebral blood flow, which increases fall risk. This interpretation is supported by our findings, as sOH was associated with falls whereas the broader OH definition was not. Future studies should consider applying both the standard and systolic OH definitions when analyzing associations with falls and other clinical outcomes to better characterize the clinical relevance of systolic blood pressure declines.

The association between recovery groups and falls from other studies shows a similar gradient to ours, where a Delayed or No Recovery from OH is frequently linked to an increased likelihood of falling. [20] Finucane et al. have demonstrated that impaired BP recovery is linked with a higher risk of injurious and unexplained falls [7]. In line with this, Petriceks et al. showed that while OH occurring immediately after standing tended to be symptomatic, it was the later-occurring OH that was significantly associated with increased fall risk [40]. Notably, the Early Recovery category observed among the majority of subjects who were classified into OH was not associated with falls. Our findings suggest that Delayed Recovery and No Recovery by sOH culminates a higher fall risk than OH, affecting a smaller percentage of subjects. Furthermore, this also suggests that recovery dynamics may be more clinically relevant than the presence of OH alone. With regard to the method applied, Mol et al. in 2022 used a sphygmomanometer to determine recovery patterns, which yielded contradictory findings [32]. They found that OH was not associated with the number of falls when recovery was defined as the BP measured at three minutes minus the one at the first minute. However, this approach may overlook important Early Recovery patterns because it only assesses recovery after the first minute.

OHTN, while not as frequent as OH, can also be found in the geriatric population. Our study showed a OHTN prevalence (15.5%) consistent with the literature, where previous studies reporting from one to 28.0% [11]. sOHTN, explored solely through the systolic criteria, is a relatively new approach, where a pre-existing prevalence for comparison could not be found. Existing research on OHTN is limited, but it has been associated with higher overall mortality and CVD. [11, 21, 41] We aimed to expand this research by evaluating OHTN in relation to falls. However, neither OHTN nor sOHTN was significantly associated with falls, and recovery time appeared considerably less relevant than in the hypotensive phenotypes. One possible explanation is that, unlike OH, OHTN is generally not accompanied by a reduction in systemic blood pressure and therefore may not impair cerebral perfusion to the same extent. Interestingly, a substantial proportion of OHTN participants demonstrated persistent blood pressure elevation after standing, due to excessive sympathetic activation upon standing [11]. Yet these prolonged hypertensive responses were not associated with increased fall risk. This contrasts with the hypotensive phenotypes and suggests that persistent blood pressure elevation and persistent blood pressure reduction may have fundamentally different clinical consequences with respect to falls. Lastly, this is the first study to evaluate BP recovery patterns in OHTN. RT appeared less clinically relevant for fall risk assessment in OHTN than in OH.

### Strengths and limitations

The primary strength and novelty of this study lies in the comprehensive evaluation of multiple orthostatic BP definitions and recovery patterns within the same cohort. Other major benefits of this study include its prospective design, which involves standardized fall assessment to minimize recall bias, and a representative sample of well-characterized, relatively high-functioning, community-dwelling older adults. Important fall-related factors, including FRIDs and s-FES-I scores, were considered, and orthostatic BP measurements followed standardized international protocols. In contrast to many previous studies, our RT categories also included the immediate post-standing period and extended to the third minute after standing. [7, 8] Nevertheless, the recovery subgroups and wide confidence intervals require cautious interpretation and confirmation in larger studies.

Several limitations should also be acknowledged. Our findings stem from data obtained from community-dwelling individuals in predominantly good health and thus should not be taken as representative of other more frail subgroups such as institutionalized, sensory-impaired or mobility-limited individuals. Secondly, although the overall sample size was large, subgroup sizes within recovery categories were smaller, limiting statistical power. OH symptoms and heart rate responses could not be analyzed due to substantial missing data.

Minor inaccuracies in BP timing may have occurred, and survivor bias cannot be excluded, as only participants attending the seven-year follow-up were included. Individuals who experienced death, hospitalization or loss to follow-up, may also have had different observation periods when it came to prospectively documenting falls. Furthermore, BP was measured intermittently using a manual sphygmomanometer rather than continuous beat-to-beat monitoring. We also recognize that although the supine-to-standing test is an established method for assessing orthostatic BP changes, it may not fully reflect everyday transitions, which often occur from sitting to standing. Thus, the findings may not be generalizable to other postural transitions. Additionally, the lack of contextual data regarding the falls, including indoor or outdoor location and timing relative to standing is another limitation. Without these details, we cannot unequivocally link the fall events to direct orthostatic blood pressure instability. Consequently, transient BP fluctuations occurring between measurement points could not be captured.

Overall, our findings suggest that delayed or absent BP recovery after standing may identify a subgroup of older adults at particularly high fall risk despite meeting standard OH criteria. Future studies should consider the entire post-standing time course, place greater emphasis on SBP changes, and further investigate the association of recovery groups with outcomes such as falls, mortality, and cardiovascular events.

## Conclusion

Our data shows an association between orthostatic hypotension and falls when using the OH definition based solely on the systolic blood pressure. When using consensus definition, OH was not associated with falls. However, when recovery time was considered, those who took more than one minute to recover and did not recover within three minutes showed a higher risk of falling, regardless of the OH definition applied. Overall, we found no association between OHTN and fall risk.

## Supplementary information

Supplementary Tables

Supplementary Tables

Graphical Abstract Orthostatic BP Fluctuations and Falls_submission

## Data Availability

The datasets used and/or analyzed during the current study are not publicly available due to data privacy and institutional restrictions. Additional information may be available from the corresponding author upon reasonable request, subject to applicable ethical and institutional approvals.

## Abbreviations

BP: Blood Pressure
SBP: Systolic Blood Pressure
DBP: Diastolic Blood Pressure
sOH: Systolic Orthostatic Hypotension
OH: Orthostatic Hypotension
sOHTN: Systolic Orthostatic Hypertension
OHTN: Orthostatic Hypertension
RT: Recovery Time
IADL: Instrumental Activities of Daily Living
ADL: Activities of Daily Living
FRIDs: Fall Risk–Increasing Drugs
s-FES-I: short version of Falls Efficacy Scale – International
HADS: Hospital Anxiety and Depression Scale
FI: Frailty Index according to Rockwood
SV: Stroke Volume
CO: Cardiac Output
