# Supplementary material for: Orthostatic blood pressure fluctuations and risk of falls in community-dwelling older adults: the role of recovery time
Source: BMC Geriatr. 2026 Oct 28;26:1360. doi: 10.1186/s12877-026-08516-1 (PMC13718094; doi:10.1186/s12877-026-08516-1)
Supplement: Supplementary file 1 [file 12877_2026_8516_MOESM1_ESM.pdf]

## Additional Files 1-4

| Additional File 1– Orthostatic Hypotension (OH) vs No Orthostatic Hypotension | | | | | | |
|---|---|---|---|---|---|---|
| | | **Instrument ranges** | **Missing** | **Total (N=430)** | **OH (n=137)** | **No OH (n=293)** |
| Age (y), median (Q1, Q3) | | | (0) | 79 (76, 82) | 78 (76, 82) | 79(76, 83) |
| **Sex (female), n (%) *** | | | **(0)** | **180 (41.9)** | **45 (32.8)** | **135 (46.1)** |
| Participants with multiple falls, n (%) | | | (0) | 58 (13.5) | 15 (10.9) | 43 (14.7) |
| Participants with 1 fall, n (%) | | | (0) | 88 (20.5) | 30 (21.9) | 58 (19.8) |
| Participants with falls overall, n (%) | | | (0) | 146 (34.0) | 45 (32.8) | 101 (34.5) |
| Falls past 12 months, n (%) | | | (4) | 124 (29.1) | 36 (26.3) | 88 (30.4) |
| Reporting symptoms, n (%) | | | (65) | | | |
| | No symptoms | | | 247 (67.7) | 83 (68.0) | 164 (67.5) |

| | | | | | |
|---|---|---|---|---|---|
| | Dizziness | | 79 (21.6) | 27 (22.1) | 52 (21.4) |
| | Nausea | | 1 (0.3) | 0 | 1 (0.4) |
| | Photopsia | | 27 (7.4) | 8 (6.6) | 19 (7.8) |
| | Other (unspecified) | | 11 (3.0) | 4 (3.3) | 4 (2.9) |
| Time to recovery of BP, n (% within column) | | (0) | | | |
| | Early recovery (<1 min) | | | 103 (75.2) | |
| | Delayed recovery (2-3 min) | | | 24 (17.5) | |
| | No recovery | | | 10 (7.3) | |
| BMI ($kg/m^2$), median (Q1, Q3) | | (1) | 27.0 (24.5, 30) | 27.6 (25, 30.3) | 26.8 (24.2, 29.6) |
| Unprompted Weight loss past year, n (%) | | (3) | 26 (6.5) | 10 (7.3) | 16 (5.8) |
| Education, n (%) | > 10 years | (6) | 124 (29.2) | 36 (27.1) | 88 (30.2) |
| | ≤ 10 years | | 300 (70.8) | 97 (72.9) | 203 (69.8) |
| Living alone, n (%) | | (0) | 145 (33.7) | 44 (32.1) | 101 (34.5) |
| Family Status, n (%) | | (0) | | | |
| | Never married | | 14 (3.3) | 2 (1.5) | 12 (4.1) |

| | | | | | |
|---|---|---|---|---|---|
| | Married | | 275 (64.0) | 92 (67.2) | 183 (62.5) |
| | Divorced | | 26 (6.0) | 9 (6.6) | 17 (5.8) |
| | Widowed | | 113 (26.3) | 33 (24.1) | 80 (27.3) |
| | Separate living | | 2 (0.5) | 1 (0.7) | 1 (0.7) |
| Apartment size ($m^2$), median, (Q1, Q3) | | (4) | 100 (82, 130) | 100 (83, 125) | 100 (82, 130) |
| SPPB[1] score, n (%) | Frail ≤ 6 | (17) | 20 (4.8) | 3 (2.3) | 17 (6.1) |
| | Pre-frail 7-9 | | 39 (9.4) | 11 (8.3) | 28 (10.0) |
| | Fit ≥10 | | 354 (85.7) | 119 (89.5) | 235 (83.9) |
| Balance score, n (%) | 0 | (7) | 8 (1.9) | 2 (1.5) | 6 (2.1) |
| | 1-2 | | 25 (5.9) | 4 (3.0) | 21 (7.3) |
| | 3-4 | | 390 (92.5) | 129 (95.5) | 261 (90.7) |
| **Gait time (s), median (Q1, Q3) [$]** | | **(5)** | **3.4 (3.0, 3.9)** | **3.3 (3.0, 3.7)** | **3.5 (3.1, 4.0)** |
| **Gait speed (m/s), median (Q1, Q3) [$]** | | **(5)** | **1.2 (1.0, 1.3)** | **1.2 (1.1, 1.3)** | **1.2 (1.0, 1.3)** |
| Gait speed < 0.8 m/s, n (%) | | (0) | 40 (9.3) | 9 (6.6) | 31 (10.6) |

| | | | | | | |
|---|---|---|---|---|---|---|
| Gait speed score, n (%) | 0 | | (1) | 4 (0.9) | 2 (1.5) | 2 (0.7) |
| | 1-2 | | | 17 (3.9) | 4 (2.9) | 13 (4.5) |
| | 3-4 | | | 408 (95.1) | 131 (95.6) | 277 (94.9) |
| Five chair rise (s), median (Q1, Q3) | | | (32) | 10.2 (8.7, 12.1) | 10.2 (8.7, 12.1) | 10.0 (8.8, 11.6) |
| Five chair rise score, n (%) | 0 | | (32) | 0 | 0 | 0 |
| | 1-2 | | | 41 (10.3) | 16 (12.1) | 25 (9.4) |
| | 3-4 | | | 357 (89.7) | 116 (87.9) | 241 (90.6) |
| Frailty Index (Rockwood), median (Q1, Q3) | | 0-1 | (102) | 0.2 (0.1, 0.2) | 0.2 (0.1, 0.2) | 0.2 (0.1, 0.2) |
| Frail with FI[2] ≥ 0.2/ Frailty (yes/no), n (%) | | | (102) | 108 (32.9) | 37 (33.3) | 71 (32.7) |
| **Handgrip strength, median (Q1, Q3) ¥** | **Men** | | **(0)** | **34 (28, 39)** | **36 (30, 40)** | **33 (27, 38)** |
| | **Women** | | **(2)** | **20 (14, 24)** | **20 (17, 25)** | **18 (14, 22)** |
| iADL[3] Index sum score, median (Q1, Q3) | | 0-8 | (2) | 1 (0, 3) | 1 (0, 2) | 1 (0, 1) |

| | | | | | |
|---|---|---|---|---|---|
| ADL[4] Index sum score, median (Q1, Q3) | 0-6 | (2) | 0 (0, 2) | 0 (0, 1) | 0 (0, 2) |
| **Short FES-I[5] score, median (Q1, Q3) [$]** | **7-28** | **(7)** | **8 (7, 10)** | **8 (7, 10)** | **8 (7, 10)** |
| HADS[7]-Depression score, median (Q1, Q3) | 0-21 | (3) | 4 (2, 6) | 4 (2, 6) | 4 (2, 6) |
| **HADS-Anxiety score, median (Q1, Q3)** | **0-21** | **(5)** | **4 (2, 6)** | **3 (1, 5)** | **4 (2, 6)** |
| HADS-Depression score ≥ 8 points, n (%) | | (0) | 59 (13.7) | 17 (12.4) | 42 (14.3) |
| **HADS-Anxiety score ≥ 8 points, n (%) *** | | **(0)** | **56 (13.0)** | **11 (8.0)** | **45 (15.4)** |
| Comorbidities | | | | | |
| Arthritis, n (%) | | (5) | 33 (7.8) | 12 (8.8) | 21 (7.3) |
| Arthrosis, n (%) | | (3) | 196 (45.9) | 57 (41.9) | 139 (47.8) |
| Cardiovascular Events, n (%) | | (0) | 120 (27.9) | 40 (29.2) | 80 (27.3) |

| | | | | | |
|---|---|---|---|---|---|
| Cardiovascular Diseases, n (%) | | | | | |
| | Heart failure | (5) | 70 (16.5) | 23 (17.0) | 47 (16.2) |
| | Stroke | (1) | 38 (8.9) | 10 (7.3) | 28 (9.6) |
| | Myocardial infarction | (4) | 41 (9.6) | 14 (10.4) | 27 (9.3) |
| | Arterial Hypertension | (2) | 252 (58.9) | 83 (61.0) | 169 (57.9) |
| **Diabetes, n (%) *** | | **(3)** | **62 (14.5)** | **29 (21.2)** | **33 (11.4)** |
| COPD, n (%) | | (2) | 16 (3.7) | 5 (3.6) | 11 (3.8) |
| Cancer, n (%) | | (1) | 110 (25.6) | 33 (24.3) | 77 (26.3) |
| Depression, n (%) | | (2) | 40 (9.3) | 11 (8.0) | 29 (10.0) |
| Renal Insufficiency, n (%) | | (12) | 27 (6.5) | 10 (7.4) | 17 (6.0) |
| High Cholesterol, n (%) | | (9) | 179 (42.5) | 58 (43.3) | 121 (42.2) |
| Circulatory Disorders (e.g. Angina Pectoris), n (%) | | (5) | 43 (10.1) | 13 (9.6) | 30 (10.3) |
| Peripheral Arterial Disease, n (%) | | (6) | 79 (18.6) | 26 (19.3) | 53 (18.3) |
| Clinostatic SBP, median (Q1, Q3) | | (0) | 134 (122, 150) | 132 (119, 149) | 135 (123, 150) |

| | | | | | |
|---|---|---|---|---|---|
| Clinostatic DBP, mean (SD) | | (0) | 75 (8.4) | 74 (8.1) | 75 (8.5) |
| Heart Rate, median (Q1, Q3) | | (0) | 71 (64, 78) | 72 (65, 79) | 71 (63,78) |
| Living Status, n (%) | | (0) | | | |
| | At home | | 420 (97.7) | 137 (100.0) | 283 (96.6) |
| | Assisted living | | 6 (1.4) | | 6 (2.0) |
| | Supervised Living Community | | 0 | | |
| | Nursing Home | | 2 (0.5) | | 2 (0.7) |
| | Other | | 2 (0.5) | | 2 (0.7) |
| Antihypertensive Treatment, n (%) | | (0) | 311 (72.3) | 101 (73.7) | 210 (71.7) |
| **Diuretic Treatment, n (%) *** | | **(0)** | **179 (41.6)** | **67 (48.9)** | **112 (38.2)** |
| Cystatin-based GFR (ml/min per 1.73 $m^2$), mean (SD) | | (4) | 54.0 (16.2) | 53.7 (16.7) | 54.2 (16.0) |

| | | | | | |
|---|---|---|---|---|---|
| Hemoglobin (g/dl), median (Q1, Q3) | | (4) | 13.7 (12.9, 14.6) | 13.8 (13.0, 14.5) | 13.7 (12.8, 14.6) |
| Hematocrit (%), median (Q1, Q3) | | (4) | 40.5 (37.9, 43.1) | 40.6 (38.2, 42.8) | 40.5 (37.8, 43.3) |
| Number of drugs, median (Q1, Q3) | | (0) | 4 (2, 6) | 4 (2.5,6.5) | 4 (2, 6) |
| **Polypharmacy (≥ 5 medications), n (%) *** | | **(0)** | **186 (43.3)** | **66 (48.2)** | **120 (41.0)** |
| No drugs taken, n (%) | | (0) | 39 (9.1) | 10 (7.3) | 29 (9.9) |
| **FRIDs[8] Medications, n (%)** | Citalopram | (36) | 4 (1.0) | 3 (2.4) | 1 (0.4) |
| | **Torasemide *** | **(36)** | **47 (11.9)** | **25 (19.7)** | **22 (8.2)** |
| | **Doxazosin** [§] | (36) | 4 (1.0) | 4 (3.1) | 0 |
| | Amitriptyline | (36) | 2 (0.5) | 1 (0.8) | 1 (0.4) |
| | **Tamsulosin *** | **(36)** | **56 (14.2)** | **29 (22.8)** | **27 (10.1)** |
| | Tramadol | (36) | 10 (2.5) | 3 (2.4) | 7 (2.6) |
| **FRIDs sum, median (Q1, Q3)** [$] | | **(36)** | **2 (1, 3)** | **2 (1, 3)** | **2 (1, 3)** |

| | | | | | |
|---|---|---|---|---|---|
| **FRIDs categorical, n (%) *** | | **(36)** | | | |
| | 0 | | **71 (18.0)** | **19 (15.0)** | **52 (19.5)** |
| | 1 | | **105 (26.6)** | **32 (25.2)** | **73 (27.3)** |
| | 2 | | **101 (25.6)** | **26 (20.5)** | **75 (28.1)** |
| | 3 | | **77 (19.5)** | **29 (22.8)** | **48 (18.0)** |
| | ≥4 | | **40 (10.1)** | **21 (16.5)** | **19 (7.1)** |
| FRIDs medication (yes/no), n (%) | | (36) | 323 (82.0) | 108 (85.0) | 215 (80.5) |
| Antidepressants, n (%) | | (36) | 27 (6.9) | 11 (8.7) | 16 (6.0) |
| Benzodiazepine use, n (%) | | (36) | 3 (0.8) | 0 | 3 (1.1) |
| Anticoagulants, n (%) | | (36) | 219 (55.6) | 77 (60.6) | 142 (53.2) |
| Statins, n (%) | | (36) | 176 (44.7) | 64 (50.4) | 112 (41.9) |
| Daily steps (median, Q1, Q3) | | (22) | 6890 (4945, 9326) | 6952 (6952, 9317) | 6844 (5020, 9447) |
| Daily steps quartiles, n (%) | | (22) | | | |
| Total | Q1 (<25th percentile) | | 101 (24.8) | 32 (24.6) | 69 (24.8) |
| | Q2 (≥25th to <50th percentile) | | 103 (25.2) | 31 (23.8) | 72 (25.9) |

| | | | | | |
|---|---|---|---|---|---|
| | Q3 (≥50th to< 75th) | | 102 (25.0) | 36 (27.7) | 66 (23.7) |
| | Q4 (≥75th percentile) | | 102 (25.0) | 31 (23.8) | 71 (25.5) |
| Men | Q1 (<25th percentile) | (13) | 56 (23.6) | 23 (26.1) | 33 (22.1) |
| | Q2 (≥25th to <50th percentile) | | 59 (24.9) | 20 (22.7) | 39 (26.2) |
| | Q3 (≥50th to< 75th) | | 61 (25.7) | 23 (26.1) | 38 (25.5) |
| | Q4 (≥75th percentile) | | 61 (25.7) | 22 (25.0) | 39 (26.2) |
| Women | Q1 (<25th percentile) | (9) | 45 (26.3) | 9 (21.4) | 36 (27.9) |
| | Q2 (≥25th to <50th percentile) | | 44 (25.7) | 11 (26.2) | 33 (25.6) |
| | Q3 (≥50th to< 75th) | | 41 (24.0) | 13 (31.0) | 28 (21.7) |
| | Q4 (≥75th percentile) | | 41 (24.0) | 9 (21.4) | 32 (24.8) |

Abbreviations: [1]SPPB- Short Physical Performance Battery Test, [2]FI – Frailty Index, [3]ADL– Activities of Daily Living, [4]iADL – instrumental Activities of Daily Living, [5]FES-I – Falls Efficacy Scale- International, , [6]HADS – Hospital Anxiety and Depression Scale, [7]FRIDs – Fall Risk Inducing Drugs

**Bold values** refer to variables with statistically significant differences between one and multiple fallers. Statistically significant differences between the two populations according to * = Chi-square, ¥ = T-test, § = Fisher Freeman Halton Exact Test or $ = Mann Whitney U test for a p-value of <0.05

| | **Additional File 2 – systolic Orthostatic Hypotension (sOH) vs No systolic Orthostatic Hypotension** | | | | | |
|---|---|---|---|---|---|---|
| | | **Instrument Ranges** | **Missing** | **Total (N= 463)** | **sOH (n=84)** | **No sOH (n=379)** |
| Age (y), median (Q1, Q3) | | | (0) | 79 (76, 83) | 79 (76, 82) | 79 (76, 83) |
| Sex (female), n (%) | | | (0) | 201 (43.4) | 31 (36.9) | 170 (44.9) |
| Participants with multiple falls, n (%) | | | (0) | 62 (13.4) | 12 (14.3) | 50 (13.2) |
| **Participants with 1 fall, n (%) *** | | | (0) | **95 (20.5)** | **26 (31.0)** | **69 (18.2)** |
| **Participants with falls overall, n (%) *** | | | (0) | **157 (33.9)** | **38 (45.2)** | **119 (31.4)** |
| Falls past 12 months, n (%) | | | (4) | 133 (29.0) | 23 (27.4) | 110 (29.3) |
| Reporting symptoms, n (%) | | | (69) | | | |
| | No symptoms | | | 265 (67.3) | 52 (72.2) | 213 (66.1) |
| | Dizziness | | | 86 (21.8) | 14 (19.4) | 72 (22.4) |
| | Nausea | | | 1 (0.3) | 0 | 1 (0.3) |

| | | | | | |
|---|---|---|---|---|---|
| | Photopsia | | 30 (7.6) | 3 (4.2) | 27 (8.4) |
| | Other (unspecified) | | 12 (3.0) | 3 (4.2) | 9 (2.8) |
| Time to recovery of BP, n (% within column) | | (0) | | | |
| | Early recovery | | | 56 (66.7) | |
| | Delayed recovery | | | 20 (23.8) | |
| | No recovery | | | 8 (9.5) | |
| BMI (kg/m$^2$), median (Q1, Q3) | | (1) | 27.0 (24.4, 29.7) | 27.1 (24.4, 29.9) | 26.9 (24.5, 30) |
| Unprompted Weight loss past year, n (%) | | (3) | 27 (6.2) | 8 (9.5) | 19 (5.3) |
| Education, n (%) | > 10 years | (6) | 133 (29.1) | 24 (29.3) | 109 (29.1) |
| | ≤ 10 years | | 324 (70.9) | 58 (70.7) | 266 (70.9) |
| Living alone, n (%) | | (0) | 161 (34.8) | 29 (34.5) | 132 (34.8) |
| Family status, n (%) | | (0) | | | |
| | Never married | | 15 (3.2) | 1 (1.2) | 14 (3.7) |
| | Married | | 292 (63.1) | 55 (65.5) | 237 (62.5) |
| | Divorced | | 29 (6.3) | 5 (6.0) | 24 (6.3) |

| | | | | | |
|---|---|---|---|---|---|
| | Widowed | | 125 (27.0) | 22 (26.2) | 103 (27.2) |
| | Separate living | | 2 (0.4) | 1 (1.2) | 1 (0.3) |
| Apartment size ($m^2$), median (Q1, Q3) | | (5) | 100 (82, 128) | 100 (76, 120) | 103 (84, 130) |
| SPPB[1] score, n (%) | Frail ≤ 6 | (18) | 20 (4.5) | 3 (3.7) | 17 (4.7) |
| | Pre-frail 7-9 | | 40 (9.0) | 7 (8.6) | 33 (9.1) |
| | Fit ≥10 | | 385 (86.5) | 71 (87.7) | 314 (86.3) |
| Balance score, n (%) | 0 | (7) | 8 (1.8) | 2 (2.4) | 6 (1.6) |
| | 1-2 | | 25 (5.5) | 2 (2.4) | 23 (6.2) |
| | 3-4 | | 423 (92.8) | 79 (95.2) | 344 (92.3) |
| Gait time (s), median (Q1, Q3) | | (6) | 3.4 (3.0, 3.9) | 3.3 (3.0, 3.7) | 3.4 (3.0, 4.0) |
| Gait speed (m/s) median (Q1, Q3) | | (6) | 1.2 (1.0, 1.3) | 1.2 (1.1, 1.3) | 1.2 (1.0, 1.3) |
| **Gait speed < 0.8 m/s, n (%) *** | | **(0)** | **41 (8.9)** | **5 (6.0)** | **36 (9.5)** |
| Gait speed score, n (%) | 0 | (2) | 4 (0.9) | 2 (2.4) | 2 (0.5) |

| | | | | | | |
|---|---|---|---|---|---|---|
| | 1-2 | | | 17 (3.7) | 2 (2.4) | 15 (4.0) |
| | 3-4 | | | 440 (95.4) | 80 (95.3) | 360 (94.5) |
| Five chair rise (s), median (Q1, Q3) | | | (32) | 10.0 (8.8, 11.6) | 10.3 (9.0, 12.9) | 10.0 (8.7, 11.5) |
| Five chair rise score, n (%) | 0 | | (32) | 0 | 0 | 0 |
| | 1-2 | | | 42 (9.8) | 15 (18.5) | 27 (7.7) |
| | 3-4 | | | 389 (90.3) | 66 (81.4) | 323 (92.3) |
| Frailty Index (Rockwood), median (Q1, Q3) | | 0-1 | (112) | 0.2 (0.1, 0.2) | 0.2 (0.1, 0.2) | 0.2 (0.1, 0.2) |
| Frail with FI[2] ≥ 0.2/ Frailty (yes/no), n (%) | | | (112) | 114 (32.5) | 24 (36.4) | 90 (31.6) |
| **Handgrip strength, median (Q1, Q3)** | Men | | (0) | 34 (28, 39) | 36 (28,39) | 34 (28,39) |
| | **Women ¥** | | **(2)** | **20 (14, 24)** | **22 (18, 26)** | **19 (14, 23)** |
| IADL[3] Index sum score, median (Q1, Q3) | | 0-8 | (2) | 1 (0, 3) | 1 (0, 3) | 1 (0, 3) |
| ADL[4] Index sum score, median (Q1, Q3) | | 0-6 | (2) | 0 (0, 2) | 0 (0, 1) | 0 (0, 2) |

| | | | | | |
|---|---|---|---|---|---|
| Short FES-I[5] score, median (Q1, Q3) | 7-28 | (7) | 8 (7, 10) | 8 (7, 10) | 8 (7, 10) |
| HADS[7]-Depression score, median (Q1, Q3) | 0-21 | (3) | 4 (2, 6) | 4 (2, 7) | 4 (2, 6) |
| **HADS-Anxiety score, median (Q1, Q3) [$]** | **0-21** | **(5)** | **4 (2, 6)** | **3 (1, 5)** | **4 (2, 6)** |
| HADS-Depression score ≥ 8 points, n (%) | | (0) | 61 (13.2) | 14 (16.7) | 47 (12.4) |
| HADS-Anxiety score ≥ 8 points, n (%) | | (0) | 60 (13.0) | 7 (8.3) | 53 (14.0) |
| | Comorbidities | | | | |
| Arthritis, n (%) | | (5) | 37 (8.1) | 9 (10.8) | 28 (7.5) |
| Arthrosis, n (%) | | (3) | 210 (45.7) | 36 (42.9) | 174 (46.3) |
| Cardiovascular Events, n (%) | | (0) | 127 (27.4) | 27 (32.1) | 100 (26.4) |
| Cardiovascular Diseases, n (%) | | | | | |
| | Heart Failure | (6) | 74 (16.2) | 14 (17.1) | 60 (16.0) |

| | | | | | |
|---|---|---|---|---|---|
| | Stroke | (1) | 41 (8.9) | 8 (9.5) | 33 (8.7) |
| | Myocardial Infarction | (4) | 42 (9.2) | 10 (12.2) | 32 (8.5) |
| | Arterial Hypertension | (2) | 272 (59.0) | 53 (63.9) | 219 (57.9) |
| **Diabetes, n (%) *** | | **(3)** | **63 (13.7)** | **20 (23.8)** | **43 (11.4)** |
| COPD, n (%) | | (2) | 18 (3.9) | 3 (3.6) | 15 (4.0) |
| Cancer, n (%) | | (1) | 114 (24.7) | 18 (21.4) | 96 (25.4) |
| Depression, n (%) | | (2) | 43 (9.3) | 8 (9.5) | 35 (9.3) |
| Renal Insufficiency, n (%) | | (12) | 29 (6.4) | 7 (8.5) | 22 (6.0) |
| High Cholesterol, n (%) | | (10) | 194 (42.8) | 41 (49.4) | 153 (41.4) |
| Circulatory Disorders, (e.g. Angina Pectoris), n (%) | | (5) | 43 (9.4) | 11 (13.4) | 32 (8.5) |
| Peripheral Arterial Disease, n (%) | | (6) | 83 (18.2) | 18 (22.0) | 65 (17.3) |
| Clinostatic SBP, median (Q1, Q3) | | (0) | 135 (122, 150) | 138 (119, 152) | 134 (123, 150) |
| **Clinostatic DBP, mean (SD)[¥]** | | **(0)** | **75.0 (8.4)** | **74.1 (8.0)** | **75.0 (8.4)** |

| | | | | | |
|---|---|---|---|---|---|
| Heart Rate median (Q1, Q3) | | (0) | 71 (64, 78) | 72 (64, 80) | 71 (64, 78) |
| Living Status, n (%) | | (0) | | | |
| | At home | | 453 (97.8) | 84 (100.0) | 369 (97.4) |
| | Assisted living | | 6 (1.3) | 0 | 6 (1.6) |
| | Supervised Living Community | | 0 | 0 | 0 |
| | Nursing Home | | 2 (0.4) | 0 | 2 (0.5) |
| | Other | | 2 (0.4) | 0 | 2 (0.5) |
| Antihypertensive Treatment, n (%) | | (0) | 328 (70.8) | 65 (77.4) | 263 (69.4) |
| **Diuretic Treatment, n (%) *** | | **(0)** | **186 (40.2)** | **44 (52.4)** | **142 (37.5)** |
| Cystatin-based GFR (ml/min per 1.73 $m^2$), mean (SD) | | (4) | 54.1 (16.1) | 52.3 (16.1) | 54.5 (16.1) |

| | | | | | |
|---|---|---|---|---|---|
| Hemoglobin (g/dl), median (Q1, Q3) | | (4) | 13.7 (12.9, 14.6) | 13.5 (12.9, 14.1) | 13.7 (12.9, 14.6) |
| Hematocrit (%), median (Q1, Q3) | | (4) | 40.5 (38.0, 43.1) | 40.2 (37.9, 42.2) | 40.6 (38.0, 43.3) |
| **Number of drugs, median (Q1, Q3) $** | | **(0)** | **4 (2.0, 6.0)** | **5 (3.0, 6.7)** | **4 (2.0, 6.0)** |
| **Polypharmacy (≥ 5 medications), n (%) *** | | **(0)** | **194 (41.9)** | **46 (54.8)** | **148 (39.1)** |
| No drugs taken, n (%) | | (0) | 46 (9.9) | 7 (8.3) | 39 (10.3) |
| FRIDs[8] Medications, n (%) | Citalopram | (43) | 4 (1.0) | 2 (2.6) | 2 (0.6) |
| | **Torasemide *** | **(43)** | **48 (11.4)** | **18 (23.4)** | **30 (8.7)** |
| | Doxazosin | (43) | 5 (1.2) | 2 (2.6) | 3 (0.9) |
| | Amitriptyline | (43) | 2 (0.5) | 1 (1.3) | 1 (0.3) |
| | **Tamsulosin *** | **(43)** | **57 (13.6)** | **17 (22.1)** | **40 (11.7)** |
| | Tramadol | (43) | 10 (2.4) | 2 (2.6) | 8 (2.3) |
| **FRIDs sum, median (Q1, Q3) $** | | **(43)** | **2 (1, 3)** | **2 (1, 3)** | **2 (1, 3)** |

| | | | | | |
|---|---|---|---|---|---|
| **FRIDs categorical, n (%) *** | | **(43)** | | | |
| | **0** | | **80 (19.0)** | **9 (11.7)** | **71 (20.7)** |
| | **1** | | **111 (26.4)** | **21 (27.3)** | **90 (26.2)** |
| | **2** | | **108 (25.7)** | **15 (19.5)** | **93 (27.1)** |
| | **3** | | **80 (19.0)** | **19 (24.7)** | **61 (17.8)** |
| | **≥4** | | **41 (9.8)** | **13 (16.9)** | **28 (8.2)** |
| FRIDs medication (yes/no), n (%) | | (43) | 340 (81.0) | 68 (88.3) | 272 (79.3) |
| Antidepressants, n (%) | | (43) | 27 (6.4) | 8 (10.4) | 19 (5.5) |
| Benzodiazepines, n (%) | | (43) | 3 (0.7) | 0 | 3 (0.9) |
| **Anticoagulants, n (%) *** | | **(43)** | **229 (54.5)** | **52 (67.5)** | **177 (51.6)** |
| **Statins, n (%) *** | | **(43)** | **184 (43.8)** | **44 (57.1)** | **140 (40.8)** |
| Daily steps (median, Q1, Q3) | | (22) | 6940 (4949, 9324) | 6806 (4895, 9009) | 7008 (5014, 9394) |
| Daily steps quartiles, n (%) | | (22) | | | |
| Total | Q1 (<25th percentile) | | 109 (24.7) | 19 (24.4) | 90 (24.8) |
| | Q2 (≥25th to <50th percentile) | | 109 (24.7) | 21 (26.9) | 88 (24.2) |

| | | | | | |
|---|---|---|---|---|---|
| | Q3 (≥50th to< 75th) | | 113 (25.6) | 20 (25.6) | 93 (25.6) |
| | Q4 (≥75th percentile) | | 110 (24.9) | 18 (23.1) | 92 (25.3) |
| Men | Q1 (<25th percentile) | (13) | 61 (24.5) | 13 (26) | 48 (24.1) |
| | Q2 (≥25th to <50th percentile) | | 61 (24.5) | 13 (26) | 48 (24.1) |
| | Q3 (≥50th to< 75th) | | 63 (25.3) | 12 (24) | 51 (25.6) |
| | Q4 (≥75th percentile) | | 64 (25.7) | 12 (24) | 52 (26.1) |
| Women | Q1 (<25th percentile) | (9) | 48 (25) | 6 (21.4) | 42 (25.6) |
| | Q2 (≥25th to <50th percentile) | | 48 (25) | 8 (28.6) | 40 (24.4) |
| | Q3 (≥50th to< 75th) | | 50 (26) | 8 (28.6) | 42 (25.6) |
| | Q4 (≥75th percentile) | | 46 (24) | 6 (21.4) | 40 (24.4) |

Abbreviations: [1]SPPB- Short Physical Performance Battery Test, [2]FI – Frailty Index, [3]ADL– Activities of Daily Living, [4]iADL – instrumental Activities of Daily Living, [5]FES-I – Falls Efficacy Scale- International, , [6]HADS – Hospital Anxiety and Depression Scale, [7]FRIDs – Fall Risk Inducing Drugs

**Bold values** refer to variables with statistically significant differences between one and multiple fallers. Statistically significant differences between the two populations according to * = Chi-square, ¥ = T-test, § = Fisher Freeman Halton Exact Test or $ = Mann Whitney U test for a p-value of <0.05

| | Additional File 3– Orthostatic Hypertension (OHTN) vs no Orthostatic Hypertension | | | | | |
|---|---|---|---|---|---|---|
| | | **Instrument Ranges** | **Missing** | **Total (N= 372)** | **OHTN (n=79)** | **No OHTN (n= 293)** |
| Age (y), median (Q1, Q3) | | | (0) | 79 (76, 83) | 79 (76, 83) | 79 (76, 83) |
| Sex (female), n (%) | | | (0) | 179 (48.1) | 44 (55.7) | 135 (46.1) |
| Participants with multiple falls, n (%) | | | (0) | 53 (14.2) | 10 (12.7) | 43 (14.7) |
| Participants with 1 fall, n (%) | | | (0) | 70 (18.8) | 12 (15.2) | 58 (19.8) |
| Participants with falls overall, n (%) | | | (0) | 123 (33.1) | 22 (29.1) | 101 (34.5) |
| Falls past 12 months, n (%) | | | (4) | 106 (28.8) | 18 (22.8) | 88 (30.4) |
| Reporting symptoms, n (%) | | | (63) | | | |
| | No symptoms | | | 209 (67.6) | 45 (68.2) | 164 (67.5) |
| | Dizziness | | | 66 (21.4) | 14 (21.2) | 52 (21.4) |
| | Nausea | | | 1 (0.3) | 0 | 1 (0.4) |
| | Photopsia | | | 24 (7.8) | 5 (7.6) | 19 (7.8) |

| | | | | | |
|---|---|---|---|---|---|
| | Other (unspecified) | | 9 (2.9) | 2 (3.0) | 7 (2.9) |
| Time to recovery of BP, n (% within column) | | (0) | | | |
| | Early recovery | | | 29 (36.7) | |
| | Delayed Recovery | | | 18 (22.8) | |
| | No recovery | | | 32 (40.5) | |
| BMI (kg/m$^2$), median (Q1, Q3) | | (1) | 26.8 (24.2, 29.6) | 27.2 (24.7, 29.3) | 26.8 (24.2, 29.6) |
| Unprompted Weight loss past year, n (%) | | (4) | 18 (5.1) | 2 (2.6) | 16 (5.8) |
| Education, n (%) | > 10 years | (2) | 111 (30) | 23 (29.1) | 88 (30.2) |
| | ≤ 10 years | | 259 (70) | 56 (70.9) | 203 (69.8) |
| Living alone, n (%) | | (0) | 136 (36.6) | 35 (44.3) | 101 (34.5) |
| Family status, n (%) | | (0) | | | |
| | Never married | | 15 (4.0) | 3 (3.8) | 12 (4.1) |
| | Married | | 225 (60.5) | 42 (53.2) | 183 (62.5) |
| | Divorced | | 22 (5.9) | 5 (6.3) | 17 (5.8) |
| | Widowed | | 109 (29.3) | 29 (36.7) | 80 (27.3) |

| | | | | | |
|---|---|---|---|---|---|
| | Separate Living | | 1 (0.3) | 0 | 1 (0.3) |
| Apartment size ($m^2$), median (Q1, Q3) | | (5) | 100 (82, 130) | 110 (82, 130) | 100 (83, 130) |
| SPPB[1] score, n (%) | Frail ≤ 6 | (14) | 20 (5.6) | 3 (3.8) | 17 (6.1) |
| | Pre-frail 7-9 | | 32 (8.9) | 4 (5.1) | 28 (10) |
| | Fit ≥10 | | 306 (85.5) | 71 (91) | 235 (83.9) |
| Balance score, n (%) | 0 | (5) | 9 (2.5) | 3 (3.8) | 6 (2.1) |
| | 1-2 | | 21 (5.8) | 0 | 21 (7.3) |
| | 3-4 | | 337 (91.9) | 76 (96.2) | 261 (90.7) |
| Gait time (s), median (Q1, Q3) | | (4) | 3.4 (3.1, 4.0) | 3.4 (3.0, 3.8) | 3.5 (3.1, 4.0) |
| Gait speed (m/s), median (Q1, Q3) | | (4) | 1.2 (1.0, 1.3) | 1.2 (1.1, 1.3) | 1.2 (1.0, 1.3) |
| Gait speed < 0.8 m/s, n (%) | | (0) | 36 (9.7) | 5 (6.3) | 31 (10.6) |
| Gait speed score, n (%) | 0 | (2) | 2 (0.5) | 0 | 2 (0.7) |
| | 1-2 | | 14 (3.8) | 1 (1.3) | 13 (4.5) |
| | 3-4 | | 354 (95.7) | 77 (98.7) | 277 (94.9) |

| | | | | | | |
|---|---|---|---|---|---|---|
| Five chair rise (s), median (Q1, Q3) | | | (30) | 10.0 (8.7, 11.6) | 9.5 (8.1, 11.3) | 10.0 (8.8, 11.6) |
| Five chair rise score, n (%) | 0 | | (30) | 0 | 0 | 0 |
| | 1-2 | | | 30 (8.8) | 5 (6.5) | 25 (9.4) |
| | 3-4 | | | 312 (91.3) | 71 (93.4) | 241 (90.6) |
| Frailty Index (Rockwood), median (Q1, Q3) | | 0-1 | (96) | 0.2 (0.1, 0.2) | 0.2 (0.1, 0.2) | 0.2 (0.1, 0.2) |
| Frail with FI[2] ≥ 0.2/ Frailty (yes/no), n (%) | | | (96) | 86 (31.2) | 15 (25.4) | 71 (32.7) |
| Handgrip strength, median (Q1, Q3) | Men | | (1) | 32 (27, 38) | 32 (27, 36) | 33 (27, 38) |
| | Women | | (2) | 18 (14, 23) | 19 (14, 25) | 18 (14, 22) |
| **iADL[3] Index sum score, median (Q1, Q3) $** | | 0-8 | **(0)** | **1 (0, 3)** | **1 (0, 2)** | **1 (0, 3)** |
| **ADL[4] Index sum score, median (Q1, Q3) $** | | 0-6 | **(0)** | **0 (0, 2)** | **0 (0, 1)** | **0 (0, 2)** |
| Short FES-I[5] score, median (Q1, Q3) | | 7-28 | (6) | 8 (7, 10) | 8 (7, 10) | 8 (7, 10) |

| | | | | | | |
|---|---|---|---|---|---|---|
| HADS[7]-Depression score, median (Q1, Q3) | | 0-21 | (1) | 4 (2, 6) | 4 (3, 6) | 4 (2, 6) |
| HADS-Anxiety score, median (Q1, Q3) | | 0-21 | (3) | 4 (2, 6) | 4 (2, 5) | 4 (2, 6) |
| HADS-Depression score ≥ 8 points, n (%) | | | (0) | 50 (13.4) | 8 (10.1) | 42 (14.3) |
| HADS-Anxiety score ≥ 8 points, n (%) | | | (0) | 52 (14) | 7 (8.9) | 45 (15.4) |
| | Comorbidities | | | | | |
| Arthritis, n (%) | | | (5) | 27 (7.4) | 6 (7.7) | 21 (7.3) |
| Arthrosis, n (%) | | | (4) | 170 (46.2) | 31 (40.3) | 139 (47.8) |
| Cardiovascular Events, n (%) | | | (0) | 95 (25.5) | 15 (19.0) | 80 (27.3) |
| Cardiovascular Diseases, n (%) | | | | | | |
| | Heart failure | | (6) | 55 (15) | 8 (10.5) | 47 (16.2) |
| | Stroke | | (1) | 32 (8.6) | 4 (5.1) | 28 (9.6) |
| | Myocardial infarction | | (2) | 33 (8.9) | 6 (7.6) | 27 (9.3) |

| | | | | |
|---|---|---|---|---|
| Arterial Hypertension | (1) | 216 (58.2) | 47 (59.5) | 169 (57.9) |
| Diabetes, n (%) | (3) | 37 (10) | 4 (5.1) | 33 (11.4) |
| COPD, n (%) | (3) | 14 (3.8) | 3 (3.8) | 11 (3.8) |
| Cancer, n (%) | (0) | 91 (24.5) | 14 (17.7) | 77 (26.3) |
| Depression, n (%) | (3) | 33 (8.9) | 4 (5.1) | 29 (10) |
| Renal Insufficiency, n (%) | (9) | 20 (5.5) | 3 (3.8) | 17 (6) |
| High Cholesterol, n (%) | (9) | 154 (42.4) | 33 (43.4) | 121 (42.2) |
| **Circulatory Disorders, (e.g. Angina Pectoris), n (%) *** | **(3)** | **32 (8.7)** | **2 (2.5)** | **30 (10.3)** |
| Peripheral Arterial Disease, n (%) | (4) | 68 (18.5) | 15 (19) | 53 (18.3) |
| **Clinostatic SBP median (Q1, Q3) $^{\$}$** | **(0)** | **137 (125, 151)** | **143 (128, 155)** | **135 (123, 150)** |
| Clinostatic DBP mean (SD) | (0) | 76 (8.4) | 77 (7.8) | 75 (8.5) |
| Heart Rate, mean (SD) | (0) | 71 (11.0) | 72 (12.1) | 71 (10.7) |
| Living Status, n (%) | (0) | | | |

| | | | | | |
|---|---|---|---|---|---|
| | At home | | 361 (97.0) | 78 (98.7) | 283 (96.6) |
| | Assisted living | | 7 (1.9) | 1 (1.3) | 6 (2.0) |
| | Supervised Living Community | | 0 | 0 | 0 |
| | Nursing Home | | 2 (0.5) | 0 | 2 (0.7) |
| | Other | | 2 (0.5) | 0 | 2 (0.7) |
| Antihypertensive Treatment, n (%) | | (0) | 261 (70.2) | 51 (64.6) | 210 (71.7) |
| **Diuretic Treatment, n (%) *** | | **(0)** | **128 (34.4)** | **16 (20.3)** | **112 (38.2)** |
| Cystatin-based GFR (ml/min per 1.73 $m^2$), mean (SD) | | (3) | 54.8 (16.1) | 57.1 (16.6) | 54.2 (16.0) |
| Hemoglobin (g/dl), median (Q1, Q3) | | (3) | 13.7 (12.9, 14.6) | 13.8 (13.1, 14.5) | 13.7 (12.8, 14.6) |
| Hematocrit (%), median (Q1, Q3) | | (3) | 40.5 (38.1, 43.5) | 41.0 (39.0, 43.7) | 40.5 (37.8, 43.3) |
| Number of drugs, median (Q1, Q3) | | (0) | 4 (2, 6) | 3 (2, 5) | 4 (2, 6) |

| | | | | | |
|---|---|---|---|---|---|
| Polypharmacy (≥ 5 medications), *n (%) | | (0) | 143 (38.4) | 23 (29.1) | 120 (41.0) |
| No drugs taken, n (%) | | (0) | 41 (11.0) | 12 (15.2) | 29 (9.9) |
| FRIDs[8] Medications, n (%) | Citalopram | (37) | 1 (0.3) | 0 | 1 (0.4) |
| | Torasemide | (37) | 27 (8.1) | 5 (7.4) | 22 (8.2) |
| | Doxazosin | (37) | 1 (0.3) | 1 (1.5) | 0 |
| | Amitriptyline | (37) | 1 (0.3) | 0 | 1 (0.4) |
| | Tamsulosin | (37) | 31 (9.3) | 4 (5.9) | 27 (10.1) |
| | Tramadol | (37) | 8 (2.4) | 1 (1.5) | 7 (2.6) |
| FRIDs sum, median (Q1, Q3) | | (37) | 2 (1, 2) | 1.5 (1, 2) | 2 (1, 3) |
| FRIDs categorical, n (%) | 0 | (37) | 66 (19.7) | 14 (20.6) | 52 (19.5) |
| | 1 | | 93 (27.8) | 20 (29.4) | 73 (27.3) |
| | 2 | | 99 (29.6) | 24 (35.3) | 75 (28.1) |
| | 3 | | 56 (16.7) | 8 (11.8) | 48 (18) |
| | ≥4 | | 21 (6.3) | 2 (2.9) | 19 (7.1) |

| | | | | | |
|---|---|---|---|---|---|
| FRIDs medications (yes/no), n (%) | | (37) | 269 (80.3) | 54 (79.4) | 215 (80.5) |
| **Antidepressants, n (%) §** | | **(38)** | **16 (4.8)** | **0** | **16 (6)** |
| Benzodiazepines, n (%) | | (38) | 3 (0.9) | 0 | 3 (1.1) |
| Anticoagulants, n (%) | | (38) | 175 (52.4) | 33 (49.3) | 142 (53.2) |
| Statins, n (%) | | (38) | 133 (39.8) | 21 (31.3) | 112 (41.9) |
| Daily steps (median, Q1, Q3) | | (17) | 6878 (5036, 9438) | 6928 (5205, 9310) | 6844 (5020, 9447) |
| Daily steps quartiles, n (%) | | (17) | | | |
| Total | Q1 (<25$^{th}$ percentile) | | 88 (24.8) | 19 (24.7) | 69 (24.8) |
| | Q2 (≥25$^{th}$ to <50$^{th}$ percentile) | | 91 (25.6) | 19 (24.7) | 72 (25.9) |
| | Q3 (≥50th to< 75th) | | 86 (24.2) | 20 (26.0) | 66 (23.7) |
| | Q4 (≥75$^{th}$ percentile) | | 90 (25.4) | 19 (24.7) | 71 (25.5) |
| Men | Q1 (<25$^{th}$ percentile) | (10) | 44 (24.0) | 11 (32.4) | 33 (22.1) |
| | Q2 (≥25$^{th}$ to <50$^{th}$ percentile) | | 48 (26.2) | 9 (26.5) | 39 (26.2) |
| | Q3 (≥50th to< 75th) | | 45 (24.6) | 7 (20.6) | 38 (25.5) |
| | Q4 (≥75$^{th}$ percentile) | | 46 (25.1) | 7 (20.6) | 39 (26.2) |

| | | | | | |
|---|---|---|---|---|---|
| Women | Q1 (<25th percentile) | (7) | 44 (25.6) | 8 (18.6) | 36 (27.9) |
| | Q2 (≥25th to <50th percentile) | | 43 (25) | 10 (23.3) | 33 (25.6) |
| | Q3 (≥50th to< 75th) | | 41 (23.8) | 13 (30.2) | 28 (21.7) |
| | Q4 (≥75th percentile) | | 44 (25.6) | 12 (27.9) | 32 (24.8) |
| | Abbreviations: [1]SPPB- Short Physical Performance Battery Test, [2]FI – Frailty Index, [3]ADL– Activities of Daily Living, [4]iADL – instrumental Activities of Daily Living, [5]FES-I – Falls Efficacy Scale- International, , [6]HADS – Hospital Anxiety and Depression Scale, [7]FRIDs – Fall Risk Inducing Drugs<br>**Bold values** refer to variables with statistically significant differences between one and multiple fallers. Statistically significant differences between the two populations according to * = Chi-square, ¥ = T-test, § = Fisher Freeman Halton Exact Test or $ = Mann Whitney U test for a p-value of <0.05 | | | | |

## Additional File 4– systolic Orthostatic Hypertension (sOHTN) vs No systolic Orthostatic Hypertension

| | | Instrument Ranges | Missing | Total (N=425) | sOHTN (n= 46) | No sOHTN (n=379) |
|---|---|---|---|---|---|---|
| Age (y), median (Q1, Q3) | | | (0) | 79 (76, 83) | 80 (77, 82) | 79 (76, 83) |
| Sex (female), n (%) | | | (0) | 193 (45.4) | 23 (50) | 170 (44.9) |
| Participants with multiple falls, n (%) | | | (0) | 56 (13.2) | 6 (13.0) | 50 (13.2) |
| Participants with 1 fall, n (%) | | | (0) | 74 (17.4) | 5 (10.9) | 69 (18.2) |
| Participants with falls overall, n (%) | | | (0) | 130 (30.6) | 11 (23.9) | 119 (31.4) |
| Falls past 12 months, n (%) | | | (4) | 119 (28.3) | 9 (19.6) | 110 (29.3) |
| Reporting symptoms, n (%) | | | (66) | | | |
| | No symptoms | | | 240 (66.9) | 27 (73.0) | 213 (66.1) |
| | Dizziness | | | 79 (22.0) | 7 (18.9) | 72 (22.4) |

| | | | | | |
|---|---|---|---|---|---|
| | Nausea | | 1 (0.3) | 0 | 1 (0.3) |
| | Photopsia | | 29 (8.1) | 2 (5.4) | 27 (8.4) |
| | Other (unspecified) | | 10 (2.8) | 1 (2.7) | 9 (2.8) |
| Time to recovery of BP, n (% within column) | | (0) | | | |
| | Early recovery | | | 18 (39.1) | |
| | Delayed recovery | | | 14 (30.4) | |
| | No recovery | | | 14 (30.4) | |
| BMI (kg/m$^2$), median (Q1, Q3) | | (1) | 27 (24.5, 29.7) | 27.4 (24.7, 29.7) | 26.9 (24.5, 29.7) |
| Unprompted Weight loss past year, n (%) | | (4) | 20 (5.0) | 1 (2.3) | 19 (5.3) |
| Education, n (%) | > 10 years | (4) | 123 (29.2) | 14 (30.4) | 109 (29.1) |
| | ≤ 10 years | | 298 (70.8) | 32 (69.6) | 266 (70.9) |
| Living alone, n (%) | | (0) | 151 (35.5) | 19 (41.3) | 132 (34.8) |
| Family status, n (%) | | (0) | | | |
| | Never married | | 16 (3.8) | 2 (4.3) | 14 (3.7) |
| | Married | | 262 (61.6) | 25 (54.3) | 237 (62.5) |

| | | | | | |
|---|---|---|---|---|---|
| | Divorced | | 26 (6.1) | 2 (4.3) | 24 (6.3) |
| | Widowed | | 120 (28.2) | 17 (37) | 103 (27.7) |
| | Separate living | | 1 (0.2) | 0 | 1 (0.3) |
| Apartment size ($m^2$), median (Q1, Q3) | | (5) | 104 (83, 130) | 108 (81, 140) | 103 (84, 130) |
| SPPB[1] score, n (%) | Frail ≤ 6 | (15) | 20 (4.9) | 3 (6.5) | 14 (4.7) |
| | Pre-frail 7-9 | | 36 (8.8) | 3 (6.5) | 33 (9.1) |
| | Fit ≥10 | | 354 (86.3) | 40 (87) | 314 (86.3) |
| Balance score, n (%) | 0 | (6) | 9 (2.1) | 3 (6.5) | 6 (1.6) |
| | 1-2 | | 23 (5.5) | 0 | 23 (6.2) |
| | 3-4 | | 387 (92.4) | 43 (93.5) | 344 (92.3) |
| Gait time (s), median (Q1, Q3) | | (4) | 3.4 (3.0, 4.0) | 3.4 (3.0, 3.8) | 3.4 (3.0, 4.0) |
| Gait speed (m/s), median (Q1, Q3) | | (4) | 1.2 (1.0, 1.3) | 1.2 (1.1, 1.3) | 1.2 (1.0, 1.3) |
| Gait speed < 0.8 m/s, n (%) | | (0) | 40 (9.4) | 4 (8.7) | 36 (9.5) |
| Gait speed score, n (%) | 0 | (2) | 2 (0.5) | 0 | 2 (0.5) |

| | | | | | | |
|---|---|---|---|---|---|---|
| | 1-2 | | | 16 (3.7) | 1 (2.2) | 15 (4.0) |
| | 3-4 | | | 405 (95.8) | 45 (97.8) | 360 (95.5) |
| Five chair rise (s), median (Q1, Q3) | | | (32) | 10.0 (8.7, 11.6) | 9.8 (7.9, 12.0) | 10.0 (8.7, 11.5) |
| Five chair rise score, n (%) | 0 | | (32) | 0 | 0 | 0 |
| | 1-2 | | | 31 (7.9) | 4 (9.3) | 27 (7.7) |
| | 3-4 | | | 362 (92.1) | 39 (90.7) | 323 (92.3) |
| Frailty Index (Rockwood), median (Q1, Q3) | | 0-1 | (104) | 0.2 (0.1, 0.2) | 0.2 (0.1, 0.2) | 0.2 (0.1, 0.2) |
| Frail with FI[2] ≥ 0.2/ Frailty (yes/no), n (%) | | | (104) | 99 (30.8) | 9 (25) | 90 (31.6) |
| Handgrip strength, median (Q1, Q3) | Men | | (1) | 34 (28, 38) | 33 (27, 38) | 34 (28, 39) |
| | Women | | (2) | 19 (14, 22) | 20 (12, 22) | 19 (14, 23) |
| IADL[3] Index sum score, median (Q1, Q3) | | 0-8 | (1) | 1 (0, 3) | 1 (0, 2) | 1 (0, 3) |
| ADL[4] Index sum score, median (Q1, Q3) | | 0-6 | (1) | 0 (0, 2) | 0 (0, 1) | 0 (0, 2) |

| | | | | | | |
|---|---|---|---|---|---|---|
| Short FES-I[5] score, median (Q1, Q3) | | 7-28 | (7) | 8 (7, 10) | 8 (7, 10) | 8 (7, 10) |
| HADS[7]-Depression score, median (Q1, Q3) | | 0-21 | (1) | 4 (2, 6) | 4 (3, 6) | 4 (2, 6) |
| HADS-Anxiety score, median (Q1, Q3) | | 0-21 | (3) | 4 (2, 6) | 3 (1, 5) | 4 (2, 6) |
| HADS-Depression score ≥ 8 points, n (%) | | | (0) | 53 (12.5) | 6 (13) | 47 (12.4) |
| HADS-Anxiety score ≥ 8 points, n (%) | | | (0) | 56 (13.2) | 3 (6.5) | 53 (14) |
| | Comorbidities | | | | | |
| Arthritis, n (%) | | | (5) | 30 (7.1) | 2 (4.4) | 28 (7.5) |
| Arthrosis, n (%) | | | (5) | 191 (45.5) | 17 (38.6) | 174 (46.3) |
| Cardiovascular Events, n (%) | | | (0) | 108 (25.4) | 8 (17.4) | 100 (26.4) |
| Cardiovascular Disease, n (%) | Heart failure | | (6) | 64 (15.3) | 4 (9.1) | 60 (16) |

| | | | | | |
|---|---|---|---|---|---|
| | Stroke | (1) | 34 (8) | 1 (2.2) | 33 (8.7) |
| | Myocardial Infarction | (2) | 37 (8.7) | 5 (10.9) | 32 (8.5) |
| | Arterial Hypertension | (1) | 246 (58) | 27 (58.7) | 219 (57.9) |
| Diabetes, n (%) | | (3) | 46 (10.9) | 3 (6.5) | 43 (11.4) |
| COPD, n (%) | | (3) | 16 (3.8) | 1 (2.2) | 15 (4.0) |
| Cancer, n (%) | | (1) | 106 (25) | 10 (21.7) | 96 (25.4) |
| Depression, n (%) | | (3) | 36 (8.5) | 1 (2.2) | 35 (9.3) |
| Renal Insufficiency, n (%) | | (10) | 23 (5.5) | 1 (2.2) | 22 (6.0) |
| High Cholesterol, n (%) | | (11) | 171 (41.3) | 18 (40.9) | 153 (41.4) |
| Circulatory Disorders (e. g. Angina Pectoris), n (%) | | (3) | 34 (8.1) | 2 (4.3) | 32 (8.5) |
| Peripheral Arterial Disease, n (%) | | (4) | 76 (18.1) | 11 (23.9) | 65 (17.3) |
| Clinostatic SBP, median (Q1, Q3) | | (0) | 135 (123, 150) | 141 (127, 153) | 134 (123, 150) |
| Clinostatic DBP, mean (SD) | | (0) | 75 (8.4) | 75.9 (8.3) | 75 (8.4) |

| | | | | | |
|---|---|---|---|---|---|
| Heart Rate, mean (SD) | | (0) | 71 (10.8) | 72.4 (13.6) | 71 (10.4) |
| Living Status, n (%) | | (0) | | | |
| | At home | | 414 (97.4) | 45 (97.8) | 369 (97.4) |
| | Assisted living | | 7 (1.6) | 1 (2.2) | 6 (1.6) |
| | Supervised Living Community | | 0 | 0 | 0 |
| | Nursing Home | | 2 (0.5) | 0 | 2 (0.5) |
| | Other | | 2 (0.5) | 0 | 2 (0.5) |
| Antihypertensive Treatment, n (%) | | (0) | 297 (69.9) | 34 (73.9) | 263 (69.4) |
| **Diuretic treatment, n (%) *** | | **(0)** | **151 (35.5)** | **9 (19.6)** | **142 (37.5)** |
| Cystatin-based GFR (ml/min per 1.73 $m^2$), mean (SD) | | (3) | 54.9 (16.3) | 58.9 (17.4) | 54.5 (16.1) |
| Hemoglobin (g/dl), median (Q1, Q3) | | (3) | 13.7 (12.9, 14.7) | 14.0 (13.3, 14.8) | 13.7 (12.9, 14.6) |

| | | | | | |
|---|---|---|---|---|---|
| Hematocrit (%), median (Q1, Q3) | | (3) | 40.7 (38.1, 43.5) | 41.7 (39.6, 44.3) | 40.6 (38.0, 43.3) |
| Number of drugs, median (Q1, Q3) | | (0) | 4 (2, 6) | 4 (2, 5) | 4 (2, 6) |
| Polypharmacy (≥ 5 medications), *n (%) | | (0) | 163 (38.4) | 15 (32.6) | 148 (39.1) |
| No drugs taken, n (%) | | (0) | 44 (10.4) | 5 (10.9) | 39 (10.3) |
| FRIDs[8] Medications, n (%) | Citalopram | (40) | 2 (0.5) | 0 | 2 (0.6) |
| | Torasemide | (40) | 34 (8.8) | 4 (9.5) | 30 (8.7) |
| | Doxazosin | (40) | 3 (0.8) | 0 | 3 (0.9) |
| | Amitriptyline | (40) | 1 (0.3) | 0 | 1 (0.3) |
| | Tamsulosin | (40) | 43 (11.2) | 3 (7.1) | 40 (11.7) |
| | Tramadol | (40) | 9 (2.3) | 1 (2.4) | 8 (2.3) |
| FRIDs sum, median (Q1, Q3) | | (40) | 2 (1, 2) | 2 (1, 2) | 2 (1, 3) |
| FRIDs categorical, n (%) | 0 | (40) | 76 (19.7) | 5 (11.9) | 71 (20.7) |
| | 1 | | 104 (27) | 14 (33.3) | 90 (26.2) |

| | | | | | |
|---|---|---|---|---|---|
| | 2 | | 110 (28.6) | 17 (40.5) | 93 (27.1) |
| | 3 | | 66 (17.1) | 5 (11.9) | 61 (17.8) |
| | ≥4 | | 29 (7.5) | 1 (2.4) | 28 (8.2) |
| FRIDs medications (yes/no), n (%) | | (40) | 309 (80.3) | 37 (88.1) | 272 (79.3) |
| Antidepressants, n (%) | | (41) | 19 (4.9) | 0 | 19 (5.5) |
| Benzodiazepines, n (%) | | (41) | 3 (0.8) | 0 | 3 (0.9) |
| Anticoagulants, n (%) | | (41) | 200 (52.1) | 23 (56.1) | 177 (51.6) |
| Statins, n (%) | | (41) | 153 (39.8) | 13 (31.7) | 140 (40.8) |
| Daily steps (median, Q1, Q3) | | (18) | 6966 (5024, 9394) | 6518 (5217, 9375) | 7008 (5014, 9394) |
| Daily steps quartiles, n (%) | | (18) | | | |
| Total | Q1 (<25$^{th}$ percentile) | | 101 (24.8) | 11 (25.0) | 90 (24.8) |
| | Q2 (≥25$^{th}$ to <50$^{th}$ percentile) | | 101 (24.8) | 13 (29.5) | 88 (24.2) |
| | Q3 (≥50th to< 75th) | | 102 (25.1) | 9 (20.5) | 93 (25.6) |
| | Q4 (≥75$^{th}$ percentile) | | 103 (25.3) | 11 (25) | 92 (25.3) |
| Men | Q1 (<25$^{th}$ percentile) | (11) | 54 (24.4) | 6 (27.3) | 48 (24.1) |

| | | | | | |
|---|---|---|---|---|---|
| | Q2 (≥25th to <50th percentile) | | 55 (24.9) | 7 (31.8) | 48 (24.1) |
| | Q3 (≥50th to< 75th) | | 56 (25.3) | 5 (22.7) | 51 (25.6) |
| | Q4 (≥75th percentile) | | 56 (25.3) | 4 (18.2) | 52 (26.1) |
| Women | Q1 (<25th percentile) | (7) | 47 (25.3) | 5 (22.7) | 42 (25.6) |
| | Q2 (≥25th to <50th percentile) | | 46 (24.7) | 6 (27.3) | 40 (24.4) |
| | Q3 (≥50th to< 75th) | | 46 (24.7) | 4 (18.2) | 42 (25.6) |
| | Q4 (≥75th percentile) | | 47 (25.3) | 7 (31.8) | 40 (24.4) |

Abbreviations: [1]SPPB- Short Physical Performance Battery Test, [2]FI – Frailty Index, [3]ADL– Activities of Daily Living, [4]iADL – instrumental Activities of Daily Living, [5]FES-I – Falls Efficacy Scale- International, [6]HADS – Hospital Anxiety and Depression Scale, [7]FRIDs – Fall Risk Inducing Drugs

**Bold values** refer to variables with statistically significant differences between one and multiple fallers. Statistically significant differences between the two populations according to * = Chi-square, ¥ = T-test, § = Fisher Freeman Halton Exact Test or $ = Mann Whitney U test for a p-value of <0.05
