# Supplementary material for: Orthostatic blood pressure fluctuations and risk of falls in community-dwelling older adults: the role of recovery time
Source: BMC Geriatr. 2026 Oct 28;26:1360. doi: 10.1186/s12877-026-08516-1 (PMC13718094; doi:10.1186/s12877-026-08516-1)
Supplement: Supplementary file 2 [file 12877_2026_8516_MOESM3_ESM.pdf]

Orthostatic blood pressure fluctuations and risk of falls in community-dwelling older adults: The Role of Recovery time.

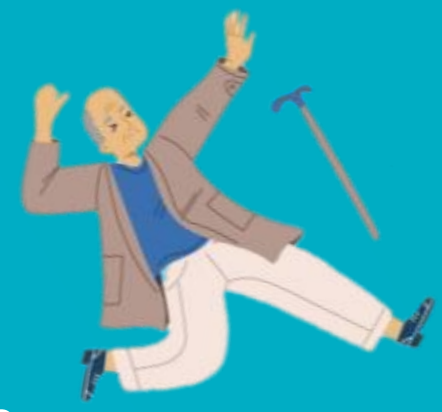

Arbieto Alvarez et al. 2026

Background

- **Orthostatic Hypotension (OH)** is prevalent in up to 44% of older adults.
- Defined as a decrease in SBP **≥ 20 mmHg** or DBP **≥ 10 mmHg within the first three minutes** of standing.
- **Systolic OH (sOH)** considers only the SBP values.

Aim of the Study

- To analyze the association between these BP fluctuations and falls, taking the Recovery time into account.

Early Recovery within 1st minute

Delayed Recovery within 2nd or 3rd minute

No Recovery during the first 3 minutes

Results

OH and Falls			
	Model[a]		Model[a]
**No OH**	Ref.	**No OH**	Ref.
**OH**	0.94 [0.57, 1.56]	**OH with Early Recovery**	0.56 [0.30, 1.01]
		**OH with Delayed Recovery**	**3.19 [1.23, 8.30]**
		**OH with No Recovery**	**4.43 [1.13, 17.30]**

sOH and Falls			
	Model[a]		Model[a]
**No sOH**	Ref.	**No sOH**	Ref.
**sOH**	**2.05 [1.17, 3.61]**	**sOH with Early Recovery**	1.23 [0.62, 2.45]
		**sOH with Delayed Recovery**	**4.97 [1.78, 13.87]**
		**sOH with No Recovery**	**5.97 [1.30, 27.38]**

[a] Adjusted for age, sex, diabetes, IADL score, hand grip strength, FRIDS, falls in the last 12 months, FES-I score, HADS-anxiety score and baseline SBP

Key Conclusions

- Only **sOH** was associated with falls.
- Models considering Recovery time from **OH/sOH** showed an increased risk for those with **Delayed** and **No Recovery**, regardless of the definition used.
