# Supplementary material for: Orthostatic blood pressure fluctuations and risk of falls in community-dwelling older adults: the role of recovery time
Source: BMC Geriatr. 2026 Oct 28;26:1360. doi: 10.1186/s12877-026-08516-1 (PMC13718094; doi:10.1186/s12877-026-08516-1)
Supplement: Supplementary file 3 [file 12877_2026_8516_MOESM2_ESM.docx]

**Orthostatic Blood Pressure Fluctuations and Risk of Falls in Community-Dwelling Older Adults: The Role of Recovery Time**

Orthostatism and falls

Valeria ARBIETO ALVAREZ (ORCID 0009-0009-2811-6236) ^a^; Clara HERTNECK MSc (ORCID 0009-0005-4693-5498) ^a^; Prof. Dr. med., MPH Dietrich ROTHENBACHER (ORCID 0000-0002-3563-2791) ^b^; Prof. Dr. med. Michael DENKINGER (ORCID 0000-0002-8097-060X) ^a, c^; Prof. Dr. med., Ph.D. Dhayana DALLMEIER (ORCID 0000-0003-3665-7023) ^a, c, d^

^a^ Institute of Geriatric Research, Ulm University Medical Center, Ulm, Germany.

^b^ Institute of Epidemiology and Medical Biometry, University Ulm, Germany.

^c^ Research Unit on Ageing, Agaplesion Bethesda Clinic Ulm, Ulm, Germany

^d^ Dept. of Epidemiology, Boston University School of Public Health, Boston, USA

**Additional Files: Additional Files 1-16**

| **Additional File 1 – Orthostatic Hypotension (OH) vs No Orthostatic Hypotension** | | | | | | |
| --- | --- | --- | --- | --- | --- | --- |
|  |  | **Instrument**  **ranges** | **Missing** | **Total (N=430)** | **OH (n=137)** | **No OH**  **(n=293)** |
| Age (y), median (Q1, Q3) |  |  | (0) | 79 (76, 82) | 78 (76, 82) | 79(76, 83) |
| **Sex (female), n (%) *** |  |  | (0) | 180 (41.9) | 45 (32.8) | 135 (46.1) |
| Participants with multiple falls, n (%) |  |  | (0) | 58 (13.5) | 15 (10.9) | 43 (14.7) |
| Participants with 1 fall, n (%) |  |  | (0) | 88 (20.5) | 30 (21.9) | 58 (19.8) |
| Participants with falls overall, n (%) |  |  | (0) | 146 (34.0) | 45 (32.8) | 101 (34.5) |
| Falls in the prior 12 months, n (%) |  |  | (4) | 124 (29.1) | 36 (26.3) | 88 (30.4) |
| Reporting symptoms, n (%) |  |  | (65) |  |  |  |
|  | No symptoms |  |  | 247 (67.7) | 83 (68.0) | 164 (67.5) |
|  | Dizziness |  |  | 79 (21.6) | 27 (22.1) | 52 (21.4) |
|  | Nausea |  |  | 1 (0.3) | 0 | 1 (0.4) |
|  | Photopsia |  |  | 27 (7.4) | 8 (6.6) | 19 (7.8) |
|  | Other (unspecified) |  |  | 11 (3.0) | 4 (3.3) | 4 (2.9) |
| Time to recovery of BP, n (% within column) |  |  | (0) |  |  |  |
|  | Early recovery (<1 min) |  |  |  | 103 (75.2) |  |
|  | Delayed recovery (2-3 min) |  |  |  | 24 (17.5) |  |
|  | No recovery |  |  |  | 10 (7.3) |  |
| BMI (kg/m^2^), median (Q1, Q3) |  |  | (1) | 27.0 (24.5, 30) | 27.6 (25, 30.3) | 26.8 (24.2, 29.6) |
| Unprompted Weight loss past year, n (%) |  |  | (3) | 26 (6.5) | 10 (7.3) | 16 (5.8) |
| Education, n (%) | > 10 years |  | (6) | 124 (29.2) | 36 (27.1) | 88 (30.2) |
|  | ≤ 10 years |  |  | 300 (70.8) | 97 (72.9) | 203 (69.8) |
| Living alone, n (%) |  |  | (0) | 145 (33.7) | 44 (32.1) | 101 (34.5) |
| Family Status, n (%) |  |  | (0) |  |  |  |
|  | Never married |  |  | 14 (3.3) | 2 (1.5) | 12 (4.1) |
|  | Married |  |  | 275 (64.0) | 92 (67.2) | 183 (62.5) |
|  | Divorced |  |  | 26 (6.0) | 9 (6.6) | 17 (5.8) |
|  | Widowed |  |  | 113 (26.3) | 33 (24.1) | 80 (27.3) |
|  | Separate living |  |  | 2 (0.5) | 1 (0.7) | 1 (0.7) |
| Apartment size (m^2^), median, (Q1, Q3) |  |  | (4) | 100 (82, 130) | 100 (83, 125) | 100 (82, 130) |
| SPPB^1^ score, n (%) | Frail ≤ 6 |  | (17) | 20 (4.8) | 3 (2.3) | 17 (6.1) |
|  | Pre-frail 7-9 |  |  | 39 (9.4) | 11 (8.3) | 28 (10.0) |
|  | Fit ≥10 |  |  | 354 (85.7) | 119 (89.5) | 235 (83.9) |
| Balance score, n (%) | 0 |  | (7) | 8 (1.9) | 2 (1.5) | 6 (2.1) |
|  | 1-2 |  |  | 25 (5.9) | 4 (3.0) | 21 (7.3) |
|  | 3-4 |  |  | 390 (92.5) | 129 (95.5) | 261 (90.7) |
| **Gait time (s), median (Q1, Q3) ^$^** |  |  | (5) | 3.4 (3.0, 3.9) | 3.3 (3.0, 3.7) | 3.5 (3.1, 4.0) |
| **Gait speed (m/s), median (Q1, Q3) ^$^** |  |  | (5) | 1.2 (1.0, 1.3) | 1.2 (1.1, 1.3) | 1.2 (1.0, 1.3) |
| Gait speed < 0.8 m/s, n (%) |  |  | (0) | 40 (9.3) | 9 (6.6) | 31 (10.6) |
| Gait speed score, n (%) | 0 |  | (1) | 4 (0.9) | 2 (1.5) | 2 (0.7) |
|  | 1-2 |  |  | 17 (3.9) | 4 (2.9) | 13 (4.5) |
|  | 3-4 |  |  | 408 (95.1) | 131 (95.6) | 277 (94.9) |
| Five chair rise (s), median (Q1, Q3) |  |  | (32) | 10.2 (8.7, 12.1) | 10.2 (8.7, 12.1) | 10.0 (8.8, 11.6) |
| Five chair rise score, n (%) | 0 |  | (32) | 0 | 0 | 0 |
|  | 1-2 |  |  | 41 (10.3) | 16 (12.1) | 25 (9.4) |
|  | 3-4 |  |  | 357 (89.7) | 116 (87.9) | 241 (90.6) |
| Frailty Index (Rockwood), median (Q1, Q3) |  | 0-1 | (102) | 0.2 (0.1, 0.2) | 0.2 (0.1, 0.2) | 0.2 (0.1, 0.2) |
| Frail with FI^2^ ≥ 0.2/ Frailty (yes/no), n (%) |  |  | (102) | 108 (32.9) | 37 (33.3) | 71 (32.7) |
| Handgrip strength, median (Q1, Q3) | **Men** |  | (0) | 34 (28, 39) | 36 (30, 40) | 33 (27, 38) |
|  | **Women** |  | (2) | 20 (14, 24) | 20 (17, 25) | 18 (14, 22) |
| iADL^3^ Index sum score, median (Q1, Q3) |  | 0-8 | (2) | 1 (0, 3) | 1 (0, 2) | 1 (0, 1) |
| ADL^4^ Index sum score, median (Q1, Q3) |  | 0-6 | (2) | 0 (0, 2) | 0 (0, 1) | 0 (0, 2) |
| Short FES-I^5^ score, median (Q1, Q3) |  | 7-28 | (7) | 8 (7, 10) | 8 (7, 10) | 8 (7, 10) |
| HADS^7^-Depression score, median (Q1, Q3) |  | 0-21 | (3) | 4 (2, 6) | 4 (2, 6) | 4 (2, 6) |
| HADS-Anxiety score, median (Q1, Q3) |  | 0-21 | (5) | 4 (2, 6) | 3 (1, 5) | 4 (2, 6) |
| HADS-Depression score ≥ 8 points, n (%) |  |  | (0) | 59 (13.7) | 17 (12.4) | 42 (14.3) |
| HADS-Anxiety score ≥ 8 points, n (%) |  |  | (0) | 56 (13.0) | 11 (8.0) | 45 (15.4) |
|  | Comorbidities | | | | | |
| Arthritis, n (%) |  |  | (5) | 33 (7.8) | 12 (8.8) | 21 (7.3) |
| Arthrosis, n (%) |  |  | (3) | 196 (45.9) | 57 (41.9) | 139 (47.8) |
| Cardiovascular Events, n (%) |  |  | (0) | 120 (27.9) | 40 (29.2) | 80 (27.3) |
| Cardiovascular Diseases, n (%) |  |  |  |  |  |  |
|  | Heart failure |  | (5) | 70 (16.5) | 23 (17.0) | 47 (16.2) |
|  | Stroke |  | (1) | 38 (8.9) | 10 (7.3) | 28 (9.6) |
|  | Myocardial infarction |  | (4) | 41 (9.6) | 14 (10.4) | 27 (9.3) |
|  | Arterial Hypertension |  | (2) | 252 (58.9) | 83 (61.0) | 169 (57.9) |
| **Diabetes, n (%) *** |  |  | (3) | 62 (14.5) | 29 (21.2) | 33 (11.4) |
| COPD, n (%) |  |  | (2) | 16 (3.7) | 5 (3.6) | 11 (3.8) |
| Cancer, n (%) |  |  | (1) | 110 (25.6) | 33 (24.3) | 77 (26.3) |
| Depression, n (%) |  |  | (2) | 40 (9.3) | 11 (8.0) | 29 (10.0) |
| Renal Insufficiency, n (%) |  |  | (12) | 27 (6.5) | 10 (7.4) | 17 (6.0) |
| High Cholesterol, n (%) |  |  | (9) | 179 (42.5) | 58 (43.3) | 121 (42.2) |
| Circulatory Disorders (e.g. Angina Pectoris), n (%) |  |  | (5) | 43 (10.1) | 13 (9.6) | 30 (10.3) |
| Peripheral Arterial Disease, n (%) |  |  | (6) | 79 (18.6) | 26 (19.3) | 53 (18.3) |
| Baseline SBP, median (Q1, Q3) |  |  | (22) | 128 (118, 139) | 129 (120, 144) | 128 (116, 138) |
| Baseline DBP, median (Q1, Q3) |  |  | (22) | 73 (69, 78) | 74 (69, 79) | 73 (69, 78) |
| Heart Rate, median (Q1, Q3) |  |  | (0) | 71 (64, 78) | 72 (65, 79) | 71 (63,78) |
| Living Status, n (%) |  |  | (0) |  |  |  |
|  | At home |  |  | 420 (97.7) | 137 (100.0) | 283 (96.6) |
|  | Assisted living |  |  | 6 (1.4) |  | 6 (2.0) |
|  | Supervised Living Community |  |  | 0 |  |  |
|  | Nursing Home |  |  | 2 (0.5) |  | 2 (0.7) |
|  | Other |  |  | 2 (0.5) |  | 2 (0.7) |
| Antihypertensive Treatment, n (%) |  |  | (0) | 311 (72.3) | 101 (73.7) | 210 (71.7) |
| Diuretic Treatment, n (%) |  |  | (0) | 179 (41.6) | 67 (48.9) | 112 (38.2) |
| Cystatin-based GFR (ml/min per 1.73 m^2^), mean (SD) |  |  | (4) | 54.0  (16.2) | 53.7  (16.7) | 54.2  (16.0) |
| Hemoglobin (g/dl), median (Q1, Q3) |  |  | (4) | 13.7  (12.9, 14.6) | 13.8  (13.0, 14.5) | 13.7  (12.8, 14.6) |
| Hematocrit (%), median (Q1, Q3) |  |  | (4) | 40.5  (37.9, 43.1) | 40.6  (38.2, 42.8) | 40.5  (37.8, 43.3) |
| Number of drugs, median (Q1, Q3) |  |  | (0) | 4 (2, 6) | 4 (2.5,6.5) | 4 (2, 6) |
| Polypharmacy (≥ 5 medications), n (%) |  |  | (0) | 186 (43.3) | 66 (48.2) | 120 (41.0) |
| No drugs taken, n (%) |  |  | (0) | 39 (9.1) | 10 (7.3) | 29 (9.9) |
| FRIDs^8^ Medications, n (%) | Citalopram |  | (36) | 4 (1.0) | 3 (2.4) | 1 (0.4) |
|  | Torasemide |  | (36) | 47 (11.9) | 25 (19.7) | 22 (8.2) |
|  | Doxazosin |  | (36) | 4 (1.0) | 4 (3.1) | 0 |
|  | Amitriptyline |  | (36) | 2 (0.5) | 1 (0.8) | 1 (0.4) |
|  | Tamsulosin |  | (36) | 56 (14.2) | 29 (22.8) | 27 (10.1) |
|  | Tramadol |  | (36) | 10 (2.5) | 3 (2.4) | 7 (2.6) |
| FRIDs sum, median (Q1, Q3) ^$^ |  |  | (36) | 2  (1, 3) | 2  (1, 3) | 2  (1, 3) |
| FRIDs categorical, n (%) |  |  | (36) |  |  |  |
|  | 0 |  |  | 71 (18.0) | 19 (15.0) | 52 (19.5) |
|  | 1 |  |  | 105 (26.6) | 32 (25.2) | 73 (27.3) |
|  | 2 |  |  | 101 (25.6) | 26 (20.5) | 75 (28.1) |
|  | 3 |  |  | 77 (19.5) | 29 (22.8) | 48 (18.0) |
|  | ≥4 |  |  | 40 (10.1) | 21 (16.5) | 19 (7.1) |
| FRIDs medication (yes/no), n (%) |  |  | (36) | 323 (82.0) | 108 (85.0) | 215 (80.5) |
| Antidepressants, n (%) |  |  | (36) | 27 (6.9) | 11 (8.7) | 16 (6.0) |
| Benzodiazepine use, n (%) |  |  | (36) | 3 (0.8) | 0 | 3 (1.1) |
| Anticoagulants, n (%) |  |  | (36) | 219 (55.6) | 77 (60.6) | 142 (53.2) |
| Statins, n (%) |  |  | (36) | 176 (44.7) | 64 (50.4) | 112 (41.9) |
| Daily steps (median, Q1, Q3) |  |  | (22) | 6890  (4945, 9326) | 6952  (6952, 9317) | 6844  (5020, 9447) |
| Daily steps quartiles, n (%) |  |  | (22) |  |  |  |
| Total | Q1 (<25^th^ percentile) |  |  | 101 (24.8) | 32 (24.6) | 69 (24.8) |
|  | Q2 (≥25^th^ to <50^th^ percentile) |  |  | 103 (25.2) | 31 (23.8) | 72 (25.9) |
|  | Q3 (≥50th to< 75th) |  |  | 102 (25.0) | 36 (27.7) | 66 (23.7) |
|  | Q4 (≥75^th^ percentile) |  |  | 102 (25.0) | 31 (23.8) | 71 (25.5) |
| Men | Q1 (<25^th^ percentile) |  | (13) | 56 (23.6) | 23 (26.1) | 33 (22.1) |
|  | Q2 (≥25^th^ to <50^th^ percentile) |  |  | 59 (24.9) | 20 (22.7) | 39 (26.2) |
|  | Q3 (≥50th to< 75th) |  |  | 61 (25.7) | 23 (26.1) | 38 (25.5) |
|  | Q4 (≥75^th^ percentile) |  |  | 61 (25.7) | 22 (25.0) | 39 (26.2) |
| Women | Q1 (<25^th^ percentile) |  | (9) | 45 (26.3) | 9 (21.4) | 36 (27.9) |
|  | Q2 (≥25^th^ to <50^th^ percentile) |  |  | 44 (25.7) | 11 (26.2) | 33 (25.6) |
|  | Q3 (≥50th to< 75th) |  |  | 41 (24.0) | 13 (31.0) | 28 (21.7) |
|  | Q4 (≥75^th^ percentile) |  |  | 41 (24.0) | 9 (21.4) | 32 (24.8) |
|  | Abbreviations: ^1^SPPB- Short Physical Performance Battery Test, ^2^FI – Frailty Index, ^3^ADL– Activities of Daily Living, ^4^iADL – instrumental Activities of Daily Living, ^5^FES-I – Falls Efficacy Scale- International, , ^6^HADS – Hospital Anxiety and Depression Scale, ^7^FRIDs – Fall Risk Inducing Drugs | | | | | |

| **Additional File 2 – systolic Orthostatic Hypotension (sOH) vs No systolic Orthostatic Hypotension** | | | | | | |
| --- | --- | --- | --- | --- | --- | --- |
|  |  | **Instrument**  **Ranges** | **Missing** | **Total (N= 463)** | **sOH**  **(n=84)** | **No sOH**  **(n=379)** |
| Age (y), median (Q1, Q3) |  |  | (0) | 79 (76, 83) | 79 (76, 82) | 79 (76, 83) |
| Sex (female), n (%) |  |  | (0) | 201 (43.4) | 31 (36.9) | 170 (44.9) |
| Participants with multiple falls, n (%) |  |  | (0) | 62 (13.4) | 12 (14.3) | 50 (13.2) |
| Participants with 1 fall, n (%) |  |  | (0) | 95 (20.5) | 26 (31.0) | **69 (18.2)** |
| Participants with falls overall, n (%) |  |  | (0) | 157 (33.9) | 38 (45.2) | **119 (31.4)** |
| Falls in the prior 12 months, n (%) |  |  | (4) | 133 (29.0) | 23 (27.4) | 110 (29.3) |
| Reporting symptoms, n (%) |  |  | (69) |  |  |  |
|  | No symptoms |  |  | 265 (67.3) | 52 (72.2) | 213 (66.1) |
|  | Dizziness |  |  | 86 (21.8) | 14 (19.4) | 72 (22.4) |
|  | Nausea |  |  | 1 (0.3) | 0 | 1 (0.3) |
|  | Photopsia |  |  | 30 (7.6) | 3 (4.2) | 27 (8.4) |
|  | Other (unspecified) |  |  | 12 (3.0) | 3 (4.2) | 9 (2.8) |
| Time to recovery of BP, n (% within column) |  |  | (0) |  |  |  |
|  | Early recovery |  |  |  | 56 (66.7) |  |
|  | Delayed recovery |  |  |  | 20 (23.8) |  |
|  | No recovery |  |  |  | 8 (9.5) |  |
| BMI (kg/m^2^), median (Q1, Q3) |  |  | (1) | 27.0 (24.4, 29.7) | 27.1 (24.4, 29.9) | 26.9 (24.5, 30) |
| Unprompted Weight loss past year, n (%) |  |  | (3) | 27 (6.2) | 8 (9.5) | 19 (5.3) |
| Education, n (%) | > 10 years |  | (6) | 133 (29.1) | 24 (29.3) | 109 (29.1) |
|  | ≤ 10 years |  |  | 324 (70.9) | 58 (70.7) | 266 (70.9) |
| Living alone, n (%) |  |  | (0) | 161 (34.8) | 29 (34.5) | 132 (34.8) |
| Family status, n (%) |  |  | (0) |  |  |  |
|  | Never married |  |  | 15 (3.2) | 1 (1.2) | 14 (3.7) |
|  | Married |  |  | 292 (63.1) | 55 (65.5) | 237 (62.5) |
|  | Divorced |  |  | 29 (6.3) | 5 (6.0) | 24 (6.3) |
|  | Widowed |  |  | 125 (27.0) | 22 (26.2) | 103 (27.2) |
|  | Separate living |  |  | 2 (0.4) | 1 (1.2) | 1 (0.3) |
| Apartment size (m^2^), median (Q1, Q3) |  |  | (5) | 100 (82, 128) | 100 (76, 120) | 103 (84, 130) |
| SPPB^1^ score, n (%) | Frail ≤ 6 |  | (18) | 20 (4.5) | 3 (3.7) | 17 (4.7) |
|  | Pre-frail 7-9 |  |  | 40 (9.0) | 7 (8.6) | 33 (9.1) |
|  | Fit ≥10 |  |  | 385 (86.5) | 71 (87.7) | 314 (86.3) |
| Balance score, n (%) | 0 |  | (7) | 8 (1.8) | 2 (2.4) | 6 (1.6) |
|  | 1-2 |  |  | 25 (5.5) | 2 (2.4) | 23 (6.2) |
|  | 3-4 |  |  | 423 (92.8) | 79 (95.2) | 344 (92.3) |
| Gait time (s), median (Q1, Q3) |  |  | (6) | 3.4 (3.0, 3.9) | 3.3 (3.0, 3.7) | 3.4 (3.0, 4.0) |
| Gait speed (m/s) median (Q1, Q3) |  |  | (6) | 1.2 (1.0, 1.3) | 1.2 (1.1, 1.3) | 1.2 (1.0, 1.3) |
| Gait speed < 0.8 m/s, n (%) |  |  | (0) | 41 (8.9) | 5 (6.0) | **36 (9.5)** |
| Gait speed score, n (%) | 0 |  | (2) | 4 (0.9) | 2 (2.4) | 2 (0.5) |
|  | 1-2 |  |  | 17 (3.7) | 2 (2.4) | 15 (4.0) |
|  | 3-4 |  |  | 440 (95.4) | 80 (95.3) | 360 (94.5) |
| Five chair rise (s), median (Q1, Q3) |  |  | (32) | 10.0 (8.8, 11.6) | 10.3 (9.0, 12.9) | 10.0 (8.7, 11.5) |
| Five chair rise score, n (%) | 0 |  | (32) | 0 | 0 | 0 |
|  | 1-2 |  |  | 42 (9.8) | 15 (18.5) | 27 (7.7) |
|  | 3-4 |  |  | 389 (90.3) | 66 (81.4) | 323 (92.3) |
| Frailty Index (Rockwood), median (Q1, Q3) |  | 0-1 | (112) | 0.2 (0.1, 0.2) | 0.2 (0.1, 0.2) | 0.2 (0.1, 0.2) |
| Frail with FI^2^ ≥ 0.2/ Frailty (yes/no), n (%) |  |  | (112) | 114 (32.5) | 24 (36.4) | 90 (31.6) |
| Handgrip strength, median (Q1, Q3) | Men |  | (0) | 34 (28, 39) | 36 (28,39) | 34 (28,39) |
|  | Women ^¥^ |  | (2) | 20 (14, 24) | 22 (18, 26) | **19 (14, 23)** |
| IADL^3^ Index sum score, median (Q1, Q3) |  | 0-8 | (2) | 1 (0, 3) | 1 (0, 3) | 1 (0, 3) |
| ADL^4^ Index sum score, median (Q1, Q3) |  | 0-6 | (2) | 0 (0, 2) | 0 (0, 1) | 0 (0, 2) |
| Short FES-I^5^ score, median (Q1, Q3) |  | 7-28 | (7) | 8 (7, 10) | 8 (7, 10) | 8 (7, 10) |
| HADS^7^-Depression score, median (Q1, Q3) |  | 0-21 | (3) | 4 (2, 6) | 4 (2, 7) | 4 (2, 6) |
| HADS-Anxiety score, median (Q1, Q3) |  | 0-21 | (5) | 4 (2, 6) | 3 (1, 5) | **4 (2, 6)** |
| HADS-Depression score ≥ 8 points, n (%) |  |  | (0) | 61 (13.2) | 14 (16.7) | 47 (12.4) |
| HADS-Anxiety score ≥ 8 points, n (%) |  |  | (0) | 60 (13.0) | 7 (8.3) | 53 (14.0) |
|  | Comorbidities | | | | | |
| Arthritis, n (%) |  |  | (5) | 37 (8.1) | 9 (10.8) | 28 (7.5) |
| Arthrosis, n (%) |  |  | (3) | 210 (45.7) | 36 (42.9) | 174 (46.3) |
| Cardiovascular Events, n (%) |  |  | (0) | 127 (27.4) | 27 (32.1) | 100 (26.4) |
| Cardiovascular Diseases, n (%) |  |  |  |  |  |  |
|  | Heart Failure |  | (6) | 74 (16.2) | 14 (17.1) | 60 (16.0) |
|  | Stroke |  | (1) | 41 (8.9) | 8 (9.5) | 33 (8.7) |
|  | Myocardial Infarction |  | (4) | 42 (9.2) | 10 (12.2) | 32 (8.5) |
|  | Arterial Hypertension |  | (2) | 272 (59.0) | 53 (63.9) | 219 (57.9) |
| Diabetes, n (%) |  |  | (3) | 63 (13.7) | 20 (23.8) | **43 (11.4)** |
| COPD, n (%) |  |  | (2) | 18 (3.9) | 3 (3.6) | 15 (4.0) |
| Cancer, n (%) |  |  | (1) | 114 (24.7) | 18 (21.4) | 96 (25.4) |
| Depression, n (%) |  |  | (2) | 43 (9.3) | 8 (9.5) | 35 (9.3) |
| Renal Insufficiency, n (%) |  |  | (12) | 29 (6.4) | 7 (8.5) | 22 (6.0) |
| High Cholesterol, n (%) |  |  | (10) | 194 (42.8) | 41 (49.4) | 153 (41.4) |
| Circulatory Disorders, (e.g. Angina Pectoris), n (%) |  |  | (5) | 43 (9.4) | 11 (13.4) | 32 (8.5) |
| Peripheral Arterial Disease, n (%) |  |  | (6) | 83 (18.2) | 18 (22.0) | 65 (17.3) |
| Baseline* SBP, median (Q1, Q3) |  |  | (22) | 129 (118, 142) | 133 (125, 148) | **128 (116, 139)** |
| Baseline DBP, median (Q1, Q3) |  |  | (22) | 73 (69, 78) | 75 (69, 79) | 73 (69, 78) |
| Heart Rate median (Q1, Q3) |  |  | (0) | 71 (64, 78) | 72 (64, 80) | 71 (64, 78) |
| Living Status, n (%) |  |  | (0) |  |  |  |
|  | At home |  |  | 453 (97.8) | 84 (100.0) | 369 (97.4) |
|  | Assisted living |  |  | 6 (1.3) | 0 | 6 (1.6) |
|  | Supervised Living Community |  |  | 0 | 0 | 0 |
|  | Nursing Home |  |  | 2 (0.4) | 0 | 2 (0.5) |
|  | Other |  |  | 2 (0.4) | 0 | 2 (0.5) |
| Antihypertensive Treatment, n (%) |  |  | (0) | 328 (70.8) | 65 (77.4) | 263 (69.4) |
| Diuretic Treatment, n (%) |  |  | (0) | 186 (40.2) | 44 (52.4) | **142 (37.5)** |
| Cystatin-based GFR (ml/min per 1.73 m^2^), mean (SD) |  |  | (4) | 54.1 (16.1) | 52.3 (16.1) | 54.5 (16.1) |
| Hemoglobin (g/dl), median (Q1, Q3) |  |  | (4) | 13.7 (12.9, 14.6) | 13.5 (12.9, 14.1) | 13.7 (12.9, 14.6) |
| Hematocrit (%), median (Q1, Q3) |  |  | (4) | 40.5 (38.0, 43.1) | 40.2 (37.9, 42.2) | 40.6 (38.0, 43.3) |
| Number of drugs, median (Q1, Q3) ^$^ |  |  | (0) | 4 (2.0, 6.0) | 5 (3.0, 6.7) | 4 (2.0, 6.0) |
| Polypharmacy (≥ 5 medications), n (%) |  |  | (0) | 194 (41.9) | 46 (54.8) | 148 (39.1) |
| No drugs taken, n (%) |  |  | (0) | 46 (9.9) | 7 (8.3) | 39 (10.3) |
| FRIDs^8^ Medications, n (%) | Citalopram |  | (43) | 4 (1.0) | 2 (2.6) | 2 (0.6) |
|  | Torasemide |  | (43) | 48 (11.4) | 18 (23.4) | **30 (8.7)** |
|  | Doxazosin |  | (43) | 5 (1.2) | 2 (2.6) | 3 (0.9) |
|  | Amitriptyline |  | (43) | 2 (0.5) | 1 (1.3) | 1 (0.3) |
|  | Tamsulosin |  | (43) | 57 (13.6) | 17 (22.1) | **40 (11.7)** |
|  | Tramadol |  | (43) | 10 (2.4) | 2 (2.6) | 8 (2.3) |
| FRIDs sum, median (Q1, Q3) ^$^ |  |  | (43) | 2 (1, 3) | 2 (1, 3) | **2 (1, 3)** |
| FRIDs categorical, n (%) |  |  | (43) |  |  |  |
|  | 0 |  |  | 80 (19.0) | 9 (11.7) | **71 (20.7)** |
|  | 1 |  |  | 111 (26.4) | 21 (27.3) | **90 (26.2)** |
|  | 2 |  |  | 108 (25.7) | 15 (19.5) | **93 (27.1)** |
|  | 3 |  |  | 80 (19.0) | 19 (24.7) | **61 (17.8)** |
|  | ≥4 |  |  | 41 (9.8) | 13 (16.9) | **28 (8.2)** |
| FRIDs medication (yes/no), n (%) |  |  | (43) | 340 (81.0) | 68 (88.3) | 272 (79.3) |
| Antidepressants, n (%) |  |  | (43) | 27 (6.4) | 8 (10.4) | 19 (5.5) |
| Benzodiazepines, n (%) |  |  | (43) | 3 (0.7) | 0 | 3 (0.9) |
| Anticoagulants, n (%) |  |  | (43) | 229 (54.5) | 52 (67.5) | **177 (51.6)** |
| Statins, n (%) |  |  | (43) | 184 (43.8) | 44 (57.1) | **140 (40.8)** |
| Daily steps (median, Q1, Q3) |  |  | (22) | 6940  (4949, 9324) | 6806  (4895, 9009) | 7008  (5014, 9394) |
| Daily steps quartiles, n (%) |  |  | (22) |  |  |  |
| Total | Q1 (<25^th^ percentile) |  |  | 109 (24.7) | 19 (24.4) | 90 (24.8) |
|  | Q2 (≥25^th^ to <50^th^ percentile) |  |  | 109 (24.7) | 21 (26.9) | 88 (24.2) |
|  | Q3 (≥50th to< 75th) |  |  | 113 (25.6) | 20 (25.6) | 93 (25.6) |
|  | Q4 (≥75^th^ percentile) |  |  | 110 (24.9) | 18 (23.1) | 92 (25.3) |
| Men | Q1 (<25^th^ percentile) |  | (13) | 61 (24.5) | 13 (26) | 48 (24.1) |
|  | Q2 (≥25^th^ to <50^th^ percentile) |  |  | 61 (24.5) | 13 (26) | 48 (24.1) |
|  | Q3 (≥50th to< 75th) |  |  | 63 (25.3) | 12 (24) | 51 (25.6) |
|  | Q4 (≥75^th^ percentile) |  |  | 64 (25.7) | 12 (24) | 52 (26.1) |
| Women | Q1 (<25^th^ percentile) |  | (9) | 48 (25) | 6 (21.4) | 42 (25.6) |
|  | Q2 (≥25^th^ to <50^th^ percentile) |  |  | 48 (25) | 8 (28.6) | 40 (24.4) |
|  | Q3 (≥50th to< 75th) |  |  | 50 (26) | 8 (28.6) | 42 (25.6) |
|  | Q4 (≥75^th^ percentile) |  |  | 46 (24) | 6 (21.4) | 40 (24.4) |
| Abbreviations: ^1^SPPB- Short Physical Performance Battery Test, ^2^FI – Frailty Index, ^3^ADL– Activities of Daily Living, ^4^iADL – instrumental Activities of Daily Living, ^5^FES-I – Falls Efficacy Scale- International, , ^6^HADS – Hospital Anxiety and Depression Scale, ^7^FRIDs – Fall Risk Inducing Drugs | | | | | | |

| **Additional File 3 – Orthostatic Hypertension (OHTN) vs no Orthostatic Hypertension** | | | | | | |
| --- | --- | --- | --- | --- | --- | --- |
|  |  | **Instrument**  **Ranges** | **Missing** | **Total (N= 372)** | **OHTN (n=79)** | **No OHTN**  **(n= 293)** |
| Age (y), median (Q1, Q3) |  |  | (0) | 79 (76, 83) | 79 (76, 83) | 79 (76, 83) |
| Sex (female), n (%) |  |  | (0) | 179 (48.1) | 44 (55.7) | 135 (46.1) |
| Participants with multiple falls, n (%) |  |  | (0) | 53 (14.2) | 10 (12.7) | 43 (14.7) |
| Participants with 1 fall, n (%) |  |  | (0) | 70 (18.8) | 12 (15.2) | 58 (19.8) |
| Participants with falls overall, n (%) |  |  | (0) | 123 (33.1) | 22 (29.1) | 101 (34.5) |
| Falls in the prior 12 months, n (%) |  |  | (4) | 106 (28.8) | 18 (22.8) | 88 (30.4) |
| Reporting symptoms, n (%) |  |  | (63) |  |  |  |
|  | No symptoms |  |  | 209 (67.6) | 45 (68.2) | 164 (67.5) |
|  | Dizziness |  |  | 66 (21.4) | 14 (21.2) | 52 (21.4) |
|  | Nausea |  |  | 1 (0.3) | 0 | 1 (0.4) |
|  | Photopsia |  |  | 24 (7.8) | 5 (7.6) | 19 (7.8) |
|  | Other (unspecified) |  |  | 9 (2.9) | 2 (3.0) | 7 (2.9) |
| Time to recovery of BP, n (% within column) |  |  | (0) |  |  |  |
|  | Early recovery |  |  |  | 29 (36.7) |  |
|  | Delayed Recovery |  |  |  | 18 (22.8) |  |
|  | No recovery |  |  |  | 32 (40.5) |  |
| BMI (kg/m^2^), median (Q1, Q3) |  |  | (1) | 26.8 (24.2, 29.6) | 27.2 (24.7, 29.3) | 26.8 (24.2, 29.6) |
| Unprompted Weight loss past year, n (%) |  |  | (4) | 18 (5.1) | 2 (2.6) | 16 (5.8) |
| Education, n (%) | > 10 years |  | (2) | 111 (30) | 23 (29.1) | 88 (30.2) |
|  | ≤ 10 years |  |  | 259 (70) | 56 (70.9) | 203 (69.8) |
| Living alone, n (%) |  |  | (0) | 136 (36.6) | 35 (44.3) | 101 (34.5) |
| Family status, n (%) |  |  | (0) |  |  |  |
|  | Never married |  |  | 15 (4.0) | 3 (3.8) | 12 (4.1) |
|  | Married |  |  | 225 (60.5) | 42 (53.2) | 183 (62.5) |
|  | Divorced |  |  | 22 (5.9) | 5 (6.3) | 17 (5.8) |
|  | Widowed |  |  | 109 (29.3) | 29 (36.7) | 80 (27.3) |
|  | Separate Living |  |  | 1 (0.3) | 0 | 1 (0.3) |
| Apartment size (m^2^), median (Q1, Q3) |  |  | (5) | 100 (82, 130) | 110 (82, 130) | 100 (83, 130) |
| SPPB^1^ score, n (%) | Frail ≤ 6 |  | (14) | 20 (5.6) | 3 (3.8) | 17 (6.1) |
|  | Pre-frail 7-9 |  |  | 32 (8.9) | 4 (5.1) | 28 (10) |
|  | Fit ≥10 |  |  | 306 (85.5) | 71 (91) | 235 (83.9) |
| Balance score, n (%) | 0 |  | (5) | 9 (2.5) | 3 (3.8) | 6 (2.1) |
|  | 1-2 |  |  | 21 (5.8) | 0 | 21 (7.3) |
|  | 3-4 |  |  | 337 (91.9) | 76 (96.2) | 261 (90.7) |
| Gait time (s), median (Q1, Q3) |  |  | (4) | 3.4 (3.1, 4.0) | 3.4 (3.0, 3.8) | 3.5 (3.1, 4.0) |
| Gait speed (m/s), median (Q1, Q3) |  |  | (4) | 1.2 (1.0, 1.3) | 1.2 (1.1, 1.3) | 1.2 (1.0, 1.3) |
| Gait speed < 0.8 m/s, n (%) |  |  | (0) | 36 (9.7) | 5 (6.3) | 31 (10.6) |
| Gait speed score, n (%) | 0 |  | (2) | 2 (0.5) | 0 | 2 (0.7) |
|  | 1-2 |  |  | 14 (3.8) | 1 (1.3) | 13 (4.5) |
|  | 3-4 |  |  | 354 (95.7) | 77 (98.7) | 277 (94.9) |
| Five chair rise (s), median (Q1, Q3) |  |  | (30) | 10.0 (8.7, 11.6) | 9.5 (8.1, 11.3) | 10.0 (8.8, 11.6) |
| Five chair rise score, n (%) | 0 |  | (30) | 0 | 0 | 0 |
|  | 1-2 |  |  | 30 (8.8) | 5 (6.5) | 25 (9.4) |
|  | 3-4 |  |  | 312 (91.3) | 71 (93.4) | 241 (90.6) |
| Frailty Index (Rockwood), median (Q1, Q3) |  | 0-1 | (96) | 0.2 (0.1, 0.2) | 0.2 (0.1, 0.2) | 0.2 (0.1, 0.2) |
| Frail with FI^2^ ≥ 0.2/ Frailty (yes/no), n (%) |  |  | (96) | 86 (31.2) | 15 (25.4) | 71 (32.7) |
| Handgrip strength, median (Q1, Q3) | Men |  | (1) | 32 (27, 38) | 32 (27, 36) | 33 (27, 38) |
|  | Women |  | (2) | 18 (14, 23) | 19 (14, 25) | 18 (14, 22) |
| iADL^3^ Index sum score, median (Q1, Q3) |  | 0-8 | (0) | 1 (0, 3) | 1 (0, 2) | 1 (0, 3) |
| ADL^4^ Index sum score, median (Q1, Q3) |  | 0-6 | (0) | 0 (0, 2) | 0 (0, 1) | 0 (0, 2) |
| Short FES-I^5^ score, median (Q1, Q3) |  | 7-28 | (6) | 8 (7, 10) | 8 (7, 10) | 8 (7, 10) |
| HADS^7^-Depression score, median (Q1, Q3) |  | 0-21 | (1) | 4 (2, 6) | 4 (3, 6) | 4 (2, 6) |
| HADS-Anxiety score, median (Q1, Q3) |  | 0-21 | (3) | 4 (2, 6) | 4 (2, 5) | 4 (2, 6) |
| HADS-Depression score ≥ 8 points, n (%) |  |  | (0) | 50 (13.4) | 8 (10.1) | 42 (14.3) |
| HADS-Anxiety score ≥ 8 points, n (%) |  |  | (0) | 52 (14) | 7 (8.9) | 45 (15.4) |
| Comorbidities | | | | | | |
| Arthritis, n (%) |  |  | (5) | 27 (7.4) | 6 (7.7) | 21 (7.3) |
| Arthrosis, n (%) |  |  | (4) | 170 (46.2) | 31 (40.3) | 139 (47.8) |
| Cardiovascular Events, n (%) |  |  | (0) | 95 (25.5) | 15 (19.0) | 80 (27.3) |
| Cardiovascular Diseases, n (%) |  |  |  |  |  |  |
|  | Heart failure |  | (6) | 55 (15) | 8 (10.5) | 47 (16.2) |
|  | Stroke |  | (1) | 32 (8.6) | 4 (5.1) | 28 (9.6) |
|  | Myocardial infarction |  | (2) | 33 (8.9) | 6 (7.6) | 27 (9.3) |
|  | Arterial Hypertension |  | (1) | 216 (58.2) | 47 (59.5) | 169 (57.9) |
| Diabetes, n (%) |  |  | (3) | 37 (10) | 4 (5.1) | 33 (11.4) |
| COPD, n (%) |  |  | (3) | 14 (3.8) | 3 (3.8) | 11 (3.8) |
| Cancer, n (%) |  |  | (0) | 91 (24.5) | 14 (17.7) | 77 (26.3) |
| Depression, n (%) |  |  | (3) | 33 (8.9) | 4 (5.1) | 29 (10) |
| Renal Insufficiency, n (%) |  |  | (9) | 20 (5.5) | 3 (3.8) | 17 (6) |
| High Cholesterol, n (%) |  |  | (9) | 154 (42.4) | 33 (43.4) | 121 (42.2) |
| Circulatory Disorders, (e.g. Angina Pectoris), n (%) |  |  | (3) | 32 (8.7) | 2 (2.5) | 30 (10.3) |
| Peripheral Arterial Disease, n (%) |  |  | (4) | 68 (18.5) | 15 (19) | 53 (18.3) |
| Baseline* SBP, median (Q1, Q3) |  |  | (22) | 127 (116, 139) | 126 (116, 144) | 128 (116, 138) |
| Baseline DBP, median (Q1, Q3) |  |  | (22) | 73 (69, 78) | 73 (69, 78) | 73 (69, 78) |
| Heart Rate, mean (SD) |  |  | (0) | 71 (11.0) | 72 (12.1) | 71 (10.7) |
| Living Status, n (%) |  |  | (0) |  |  |  |
|  | At home |  |  | 361 (97.0) | 78 (98.7) | 283 (96.6) |
|  | Assisted living |  |  | 7 (1.9) | 1 (1.3) | 6 (2.0) |
|  | Supervised Living Community |  |  | 0 | 0 | 0 |
|  | Nursing Home |  |  | 2 (0.5) | 0 | 2 (0.7) |
|  | Other |  |  | 2 (0.5) | 0 | 2 (0.7) |
| Antihypertensive Treatment, n (%) |  |  | (0) | 261 (70.2) | 51 (64.6) | 210 (71.7) |
| Diuretic Treatment, n (%) |  |  | (0) | 128 (34.4) | 16 (20.3) | 112 (38.2) |
| Cystatin-based GFR (ml/min per 1.73 m^2^), mean (SD) |  |  | (3) | 54.8 (16.1) | 57.1 (16.6) | 54.2 (16.0) |
| Hemoglobin (g/dl), median (Q1, Q3) |  |  | (3) | 13.7 (12.9, 14.6) | 13.8 (13.1, 14.5) | 13.7 (12.8, 14.6) |
| Hematocrit (%), median (Q1, Q3) |  |  | (3) | 40.5 (38.1, 43.5) | 41.0 (39.0, 43.7) | 40.5 (37.8, 43.3) |
| Number of drugs, median (Q1, Q3) |  |  | (0) | 4 (2, 6) | 3 (2, 5) | 4 (2, 6) |
| Polypharmacy (≥ 5 medications), *n (%) |  |  | (0) | 143 (38.4) | 23 (29.1) | 120 (41.0) |
| No drugs taken, n (%) |  |  | (0) | 41 (11.0) | 12 (15.2) | 29 (9.9) |
| FRIDs^8^ Medications, n (%) | Citalopram |  | (37) | 1 (0.3) | 0 | 1 (0.4) |
|  | Torasemide |  | (37) | 27 (8.1) | 5 (7.4) | 22 (8.2) |
|  | Doxazosin |  | (37) | 1 (0.3) | 1 (1.5) | 0 |
|  | Amitriptyline |  | (37) | 1 (0.3) | 0 | 1 (0.4) |
|  | Tamsulosin |  | (37) | 31 (9.3) | 4 (5.9) | 27 (10.1) |
|  | Tramadol |  | (37) | 8 (2.4) | 1 (1.5) | 7 (2.6) |
| FRIDs sum, median (Q1, Q3) |  |  | (37) | 2 (1, 2) | 1.5 (1, 2) | 2 (1, 3) |
| FRIDs categorical, n (%) | 0 |  | (37) | 66 (19.7) | 14 (20.6) | 52 (19.5) |
|  | 1 |  |  | 93 (27.8) | 20 (29.4) | 73 (27.3) |
|  | 2 |  |  | 99 (29.6) | 24 (35.3) | 75 (28.1) |
|  | 3 |  |  | 56 (16.7) | 8 (11.8) | 48 (18) |
|  | ≥4 |  |  | 21 (6.3) | 2 (2.9) | 19 (7.1) |
| FRIDs medications (yes/no), n (%) |  |  | (37) | 269 (80.3) | 54 (79.4) | 215 (80.5) |
| Antidepressants, n (%) ^§^ |  |  | (38) | 16 (4.8) | 0 | 16 (6) |
| Benzodiazepines, n (%) |  |  | (38) | 3 (0.9) | 0 | 3 (1.1) |
| Anticoagulants, n (%) |  |  | (38) | 175 (52.4) | 33 (49.3) | 142 (53.2) |
| Statins, n (%) |  |  | (38) | 133 (39.8) | 21 (31.3) | 112 (41.9) |
| Daily steps (median, Q1, Q3) |  |  | (17) | 6878  (5036, 9438) | 6928  (5205, 9310) | 6844  (5020, 9447) |
| Daily steps quartiles, n (%) |  |  | (17) |  |  |  |
| Total | Q1 (<25^th^ percentile) |  |  | 88 (24.8) | 19 (24.7) | 69 (24.8) |
|  | Q2 (≥25^th^ to <50^th^ percentile) |  |  | 91 (25.6) | 19 (24.7) | 72 (25.9) |
|  | Q3 (≥50th to< 75th) |  |  | 86 (24.2) | 20 (26.0) | 66 (23.7) |
|  | Q4 (≥75^th^ percentile) |  |  | 90 (25.4) | 19 (24.7) | 71 (25.5) |
| Men | Q1 (<25^th^ percentile) |  | (10) | 44 (24.0) | 11 (32.4) | 33 (22.1) |
|  | Q2 (≥25^th^ to <50^th^ percentile) |  |  | 48 (26.2) | 9 (26.5) | 39 (26.2) |
|  | Q3 (≥50th to< 75th) |  |  | 45 (24.6) | 7 (20.6) | 38 (25.5) |
|  | Q4 (≥75^th^ percentile) |  |  | 46 (25.1) | 7 (20.6) | 39 (26.2) |
| Women | Q1 (<25^th^ percentile) |  | (7) | 44 (25.6) | 8 (18.6) | 36 (27.9) |
|  | Q2 (≥25^th^ to <50^th^ percentile) |  |  | 43 (25) | 10 (23.3) | 33 (25.6) |
|  | Q3 (≥50th to< 75th) |  |  | 41 (23.8) | 13 (30.2) | 28 (21.7) |
|  | Q4 (≥75^th^ percentile) |  |  | 44 (25.6) | 12 (27.9) | 32 (24.8) |
| Abbreviations: ^1^SPPB- Short Physical Performance Battery Test, ^2^FI – Frailty Index, ^3^ADL– Activities of Daily Living, ^4^iADL – instrumental Activities of Daily Living, ^5^FES-I – Falls Efficacy Scale- International, , ^6^HADS – Hospital Anxiety and Depression Scale, ^7^FRIDs – Fall Risk Inducing Drugs | | | | | | |

| **Additional File 4 – systolic Orthostatic Hypertension (sOHTN) vs No systolic Orthostatic Hypertension** | | | | | | |
| --- | --- | --- | --- | --- | --- | --- |
|  |  | **Instrument**  **Ranges** | **Missing** | **Total (N=425)** | **sOHTN**  **(n= 46)** | **No sOHTN**  **(n=379)** |
| Age (y), median (Q1, Q3) |  |  | (0) | 79 (76, 83) | 80 (77, 82) | 79 (76, 83) |
| Sex (female), n (%) |  |  | (0) | 193 (45.4) | 23 (50) | 170 (44.9) |
| Participants with multiple falls, n (%) |  |  | (0) | 56 (13.2) | 6 (13.0) | 50 (13.2) |
| Participants with 1 fall, n (%) |  |  | (0) | 74 (17.4) | 5 (10.9) | 69 (18.2) |
| Participants with falls overall, n (%) |  |  | (0) | 130 (30.6) | 11 (23.9) | 119 (31.4) |
| Falls in the prior 12 months, n (%) |  |  | (4) | 119 (28.3) | 9 (19.6) | 110 (29.3) |
| Reporting symptoms, n (%) |  |  | (66) |  |  |  |
|  | No symptoms |  |  | 240 (66.9) | 27 (73.0) | 213 (66.1) |
|  | Dizziness |  |  | 79 (22.0) | 7 (18.9) | 72 (22.4) |
|  | Nausea |  |  | 1 (0.3) | 0 | 1 (0.3) |
|  | Photopsia |  |  | 29 (8.1) | 2 (5.4) | 27 (8.4) |
|  | Other (unspecified) |  |  | 10 (2.8) | 1 (2.7) | 9 (2.8) |
| Time to recovery of BP, n (% within column) |  |  | (0) |  |  |  |
|  | Early recovery |  |  |  | 18 (39.1) |  |
|  | Delayed recovery |  |  |  | 14 (30.4) |  |
|  | No recovery |  |  |  | 14 (30.4) |  |
| BMI (kg/m^2^), median (Q1, Q3) |  |  | (1) | 27 (24.5, 29.7) | 27.4 (24.7, 29.7) | 26.9 (24.5, 29.7) |
| Unprompted Weight loss past year, n (%) |  |  | (4) | 20 (5.0) | 1 (2.3) | 19 (5.3) |
| Education, n (%) | > 10 years |  | (4) | 123 (29.2) | 14 (30.4) | 109 (29.1) |
|  | ≤ 10 years |  |  | 298 (70.8) | 32 (69.6) | 266 (70.9) |
| Living alone, n (%) |  |  | (0) | 151 (35.5) | 19 (41.3) | 132 (34.8) |
| Family status, n (%) |  |  | (0) |  |  |  |
|  | Never married |  |  | 16 (3.8) | 2 (4.3) | 14 (3.7) |
|  | Married |  |  | 262 (61.6) | 25 (54.3) | 237 (62.5) |
|  | Divorced |  |  | 26 (6.1) | 2 (4.3) | 24 (6.3) |
|  | Widowed |  |  | 120 (28.2) | 17 (37) | 103 (27.7) |
|  | Separate living |  |  | 1 (0.2) | 0 | 1 (0.3) |
| Apartment size (m^2^), median (Q1, Q3) |  |  | (5) | 104 (83, 130) | 108 (81, 140) | 103 (84, 130) |
| SPPB^1^ score, n (%) | Frail ≤ 6 |  | (15) | 20 (4.9) | 3 (6.5) | 14 (4.7) |
|  | Pre-frail 7-9 |  |  | 36 (8.8) | 3 (6.5) | 33 (9.1) |
|  | Fit ≥10 |  |  | 354 (86.3) | 40 (87) | 314 (86.3) |
| Balance score, n (%) | 0 |  | (6) | 9 (2.1) | 3 (6.5) | 6 (1.6) |
|  | 1-2 |  |  | 23 (5.5) | 0 | 23 (6.2) |
|  | 3-4 |  |  | 387 (92.4) | 43 (93.5) | 344 (92.3) |
| Gait time (s), median (Q1, Q3) |  |  | (4) | 3.4 (3.0, 4.0) | 3.4 (3.0, 3.8) | 3.4 (3.0, 4.0) |
| Gait speed (m/s), median (Q1, Q3) |  |  | (4) | 1.2 (1.0, 1.3) | 1.2 (1.1, 1.3) | 1.2 (1.0, 1.3) |
| Gait speed < 0.8 m/s, n (%) |  |  | (0) | 40 (9.4) | 4 (8.7) | 36 (9.5) |
| Gait speed score, n (%) | 0 |  | (2) | 2 (0.5) | 0 | 2 (0.5) |
|  | 1-2 |  |  | 16 (3.7) | 1 (2.2) | 15 (4.0) |
|  | 3-4 |  |  | 405 (95.8) | 45 (97.8) | 360 (95.5) |
| Five chair rise (s), median (Q1, Q3) |  |  | (32) | 10.0 (8.7, 11.6) | 9.8 (7.9, 12.0) | 10.0 (8.7, 11.5) |
| Five chair rise score, n (%) | 0 |  | (32) | 0 | 0 | 0 |
|  | 1-2 |  |  | 31 (7.9) | 4 (9.3) | 27 (7.7) |
|  | 3-4 |  |  | 362 (92.1) | 39 (90.7) | 323 (92.3) |
| Frailty Index (Rockwood), median (Q1, Q3) |  | 0-1 | (104) | 0.2 (0.1, 0.2) | 0.2 (0.1, 0.2) | 0.2 (0.1, 0.2) |
| Frail with FI^2^ ≥ 0.2/ Frailty (yes/no), n (%) |  |  | (104) | 99 (30.8) | 9 (25) | 90 (31.6) |
| Handgrip strength, median (Q1, Q3) | Men |  | (1) | 34 (28, 38) | 33 (27, 38) | 34 (28, 39) |
|  | Women |  | (2) | 19 (14, 22) | 20 (12, 22) | 19 (14, 23) |
| IADL^3^ Index sum score, median (Q1, Q3) |  | 0-8 | (1) | 1 (0, 3) | 1 (0, 2) | 1 (0, 3) |
| ADL^4^ Index sum score, median (Q1, Q3) |  | 0-6 | (1) | 0 (0, 2) | 0 (0, 1) | 0 (0, 2) |
| Short FES-I^5^ score, median (Q1, Q3) |  | 7-28 | (7) | 8 (7, 10) | 8 (7, 10) | 8 (7, 10) |
| HADS^7^-Depression score, median (Q1, Q3) |  | 0-21 | (1) | 4 (2, 6) | 4 (3, 6) | 4 (2, 6) |
| HADS-Anxiety score, median (Q1, Q3) |  | 0-21 | (3) | 4 (2, 6) | 3 (1, 5) | 4 (2, 6) |
| HADS-Depression score ≥ 8 points, n (%) |  |  | (0) | 53 (12.5) | 6 (13) | 47 (12.4) |
| HADS-Anxiety score ≥ 8 points, n (%) |  |  | (0) | 56 (13.2) | 3 (6.5) | 53 (14) |
|  | Comorbidities | | | | | |
| Arthritis, n (%) |  |  | (5) | 30 (7.1) | 2 (4.4) | 28 (7.5) |
| Arthrosis, n (%) |  |  | (5) | 191 (45.5) | 17 (38.6) | 174 (46.3) |
| Cardiovascular Events, n (%) |  |  | (0) | 108 (25.4) | 8 (17.4) | 100 (26.4) |
| Cardiovascular Disease, n (%) |  |  |  |  |  |  |
|  | Heart failure |  | (6) | 64 (15.3) | 4 (9.1) | 60 (16) |
|  | Stroke |  | (1) | 34 (8) | 1 (2.2) | 33 (8.7) |
|  | Myocardial Infarction |  | (2) | 37 (8.7) | 5 (10.9) | 32 (8.5) |
|  | Arterial Hypertension |  | (1) | 246 (58) | 27 (58.7) | 219 (57.9) |
| Diabetes, n (%) |  |  | (3) | 46 (10.9) | 3 (6.5) | 43 (11.4) |
| COPD, n (%) |  |  | (3) | 16 (3.8) | 1 (2.2) | 15 (4.0) |
| Cancer, n (%) |  |  | (1) | 106 (25) | 10 (21.7) | 96 (25.4) |
| Depression, n (%) |  |  | (3) | 36 (8.5) | 1 (2.2) | 35 (9.3) |
| Renal Insufficiency, n (%) |  |  | (10) | 23 (5.5) | 1 (2.2) | 22 (6.0) |
| High Cholesterol, n (%) |  |  | (11) | 171 (41.3) | 18 (40.9) | 153 (41.4) |
| Circulatory Disorders (e. g. Angina Pectoris), n (%) |  |  | (3) | 34 (8.1) | 2 (4.3) | 32 (8.5) |
| Peripheral Arterial Disease, n (%) |  |  | (4) | 76 (18.1) | 11 (23.9) | 65 (17.3) |
| Baseline SBP, median (Q1, Q3) |  |  | (22) | 126 (116, 138) | 122 (116, 130) | 128 (116, 139) |
| Baseline DBP, median (Q1, Q3) |  |  | (22) | 73 (69, 78) | 73 (68, 77) | 73 (69, 78) |
| Heart Rate, mean (SD) |  |  | (0) | 71 (10.8) | 72.4 (13.6) | 71 (10.4) |
| Living Status, n (%) |  |  | (0) |  |  |  |
|  | At home |  |  | 414 (97.4) | 45 (97.8) | 369 (97.4) |
|  | Assisted living |  |  | 7 (1.6) | 1 (2.2) | 6 (1.6) |
|  | Supervised Living Community |  |  | 0 | 0 | 0 |
|  | Nursing Home |  |  | 2 (0.5) | 0 | 2 (0.5) |
|  | Other |  |  | 2 (0.5) | 0 | 2 (0.5) |
| Antihypertensive Treatment, n (%) |  |  | (0) | 297 (69.9) | 34 (73.9) | 263 (69.4) |
| Diuretic treatment, n (%) |  |  | (0) | 151 (35.5) | 9 (19.6) | 142 (37.5) |
| Cystatin-based GFR (ml/min per 1.73 m^2^), mean (SD) |  |  | (3) | 54.9 (16.3) | 58.9 (17.4) | 54.5 (16.1) |
| Hemoglobin (g/dl), median (Q1, Q3) |  |  | (3) | 13.7 (12.9, 14.7) | 14.0 (13.3, 14.8) | 13.7 (12.9, 14.6) |
| Hematocrit (%), median (Q1, Q3) |  |  | (3) | 40.7 (38.1, 43.5) | 41.7 (39.6, 44.3) | 40.6 (38.0, 43.3) |
| Number of drugs, median (Q1, Q3) |  |  | (0) | 4 (2, 6) | 4 (2, 5) | 4 (2, 6) |
| Polypharmacy (≥ 5 medications), *n (%) |  |  | (0) | 163 (38.4) | 15 (32.6) | 148 (39.1) |
| No drugs taken, n (%) |  |  | (0) | 44 (10.4) | 5 (10.9) | 39 (10.3) |
| FRIDs^8^ Medications, n (%) | Citalopram |  | (40) | 2 (0.5) | 0 | 2 (0.6) |
|  | Torasemide |  | (40) | 34 (8.8) | 4 (9.5) | 30 (8.7) |
|  | Doxazosin |  | (40) | 3 (0.8) | 0 | 3 (0.9) |
|  | Amitriptyline |  | (40) | 1 (0.3) | 0 | 1 (0.3) |
|  | Tamsulosin |  | (40) | 43 (11.2) | 3 (7.1) | 40 (11.7) |
|  | Tramadol |  | (40) | 9 (2.3) | 1 (2.4) | 8 (2.3) |
| FRIDs sum, median (Q1, Q3) |  |  | (40) | 2 (1, 2) | 2 (1, 2) | 2 (1, 3) |
| FRIDs categorical, n (%) | 0 |  | (40) | 76 (19.7) | 5 (11.9) | 71 (20.7) |
|  | 1 |  |  | 104 (27) | 14 (33.3) | 90 (26.2) |
|  | 2 |  |  | 110 (28.6) | 17 (40.5) | 93 (27.1) |
|  | 3 |  |  | 66 (17.1) | 5 (11.9) | 61 (17.8) |
|  | ≥4 |  |  | 29 (7.5) | 1 (2.4) | 28 (8.2) |
| FRIDs medications (yes/no), n (%) |  |  | (40) | 309 (80.3) | 37 (88.1) | 272 (79.3) |
| Antidepressants, n (%) |  |  | (41) | 19 (4.9) | 0 | 19 (5.5) |
| Benzodiazepines, n (%) |  |  | (41) | 3 (0.8) | 0 | 3 (0.9) |
| Anticoagulants, n (%) |  |  | (41) | 200 (52.1) | 23 (56.1) | 177 (51.6) |
| Statins, n (%) |  |  | (41) | 153 (39.8) | 13 (31.7) | 140 (40.8) |
| Daily steps (median, Q1, Q3) |  |  | (18) | 6966  (5024, 9394) | 6518  (5217, 9375) | 7008  (5014, 9394) |
| Daily steps quartiles, n (%) |  |  | (18) |  |  |  |
| Total | Q1 (<25^th^ percentile) |  |  | 101 (24.8) | 11 (25.0) | 90 (24.8) |
|  | Q2 (≥25^th^ to <50^th^ percentile) |  |  | 101 (24.8) | 13 (29.5) | 88 (24.2) |
|  | Q3 (≥50th to< 75th) |  |  | 102 (25.1) | 9 (20.5) | 93 (25.6) |
|  | Q4 (≥75^th^ percentile) |  |  | 103 (25.3) | 11 (25) | 92 (25.3) |
| Men | Q1 (<25^th^ percentile) |  | (11) | 54 (24.4) | 6 (27.3) | 48 (24.1) |
|  | Q2 (≥25^th^ to <50^th^ percentile) |  |  | 55 (24.9) | 7 (31.8) | 48 (24.1) |
|  | Q3 (≥50th to< 75th) |  |  | 56 (25.3) | 5 (22.7) | 51 (25.6) |
|  | Q4 (≥75^th^ percentile) |  |  | 56 (25.3) | 4 (18.2) | 52 (26.1) |
| Women | Q1 (<25^th^ percentile) |  | (7) | 47 (25.3) | 5 (22.7) | 42 (25.6) |
|  | Q2 (≥25^th^ to <50^th^ percentile) |  |  | 46 (24.7) | 6 (27.3) | 40 (24.4) |
|  | Q3 (≥50th to< 75th) |  |  | 46 (24.7) | 4 (18.2) | 42 (25.6) |
|  | Q4 (≥75^th^ percentile) |  |  | 47 (25.3) | 7 (31.8) | 40 (24.4) |
| Abbreviations: ^1^SPPB- Short Physical Performance Battery Test, ^2^FI – Frailty Index, ^3^ADL– Activities of Daily Living, ^4^iADL – instrumental Activities of Daily Living, ^5^FES-I – Falls Efficacy Scale- International, ^6^HADS – Hospital Anxiety and Depression Scale, ^7^FRIDs – Fall Risk Inducing Drugs | | | | | | |

**Additional File 5: Logistic regression between OH^1^ and falls (n=378, 128 falls) – with covariates OR and CI 95%**

|  | Participants with falls/n | Model^a^ | Model^b^ | Recovery Categories | Participants with falls/n | Model^a^ | Model^b^ |
| --- | --- | --- | --- | --- | --- | --- | --- |
| No OH | 89/255 | Ref. | Ref | No OH | 89/255 | Ref. | Ref |
| OH | 39/123 | 0.96 [0.58, 1.58] | 0.94  [0.57, 1.56] | OH with Early Recovery | 20/91 | 0.57  [0.31, 1.03] | 0.56  [0.30, 1.01] |
|  |  |  |  | OH with Delayed Recovery | 13/22 | 3.11  [1.21, 8.01] | **3.19**  **[1.23, 8.30]** |
|  |  |  |  | OH with NoRecovery | 6/10 | 4.04  [1.07, 15.26] | **4.43**  **[1.13, 17.30]** |
| Covariates |  |  |  |  |  |  |  |
| Sex |  | 0.99  [0.52, 1.88] | 0.93  [0.47, 1.82] |  |  | 1.05  [0.54, 2.01] | 1.02  [ 0.51, 2.03] |
| Age |  | 0.96  [0.91, 1.02] | 0.96  [0.91, 1.01] |  |  | 0.96  [0.91, 1.02] | 0.96  [0.91, 1.02] |
| Diabetes |  | 1.05  [0.54, 2.04] | 1.11  [0.57, 2.17] |  |  | 0.96  [0.49, 1.90] | 1.01  [0.51, 2.02] |
| IADL |  | **1.10**  **[1.00, 1.20]** | 1.09  [0.99, 1.20] |  |  | **1.11**  **[1.02, 1.22]** | **1.11**  **[1.00, 1.23]** |
| Hand Grip Strength |  | 1.00  [0.97, 1.03] | 1.00  [0.96, 1.03] |  |  | 1.01  [0.97, 1.04] | 1.01  [0.97, 1.04 ] |
| FRIDs |  | 1.05  [0.87, 1.27] | 1.06  [0.87, 1.28] |  |  | 1.01  [0.83, 1.23] | 1.02  [0.84, 1.25] |
| HADS-A score |  | 1.35  [0.71, 2,56] | 1.31  [0.67, 2.57] |  |  | 1.46  [0.76, 2.82] | 1.46  [0.73, 2.90] |
| sFES-I score |  | 1.05  [0.95, 1.15] | 1.04  [ 0.94, 1.15] |  |  | 1.04  [0.94, 1.14] | 1.03  [0.92, 1.14] |
| Falls in the prior 12 months |  | **1.92**  **[1.21, 3.05]** | **1.95**  **[1.21, 3.14]** |  |  | **2.10**  **[1.30, 3.40]** | **2.16**  **[1.32, 3.55]** |
| Baseline SBP |  |  | 1.00  [0.99, 1.02 ] |  |  |  | 1.00  [0.98, 1.01] |
| ^a^Adjusted for age, sex, diabetes, instrumental Activities of Daily Living (iADL) score, hand grip strength, Fall Risk-Increasing Drugs (FRIDs), falls in the prior 12 months, Falls Efficacy Scale-International (FES-I) score, Hospital Anxiety and Depression Scale (HADS)-anxiety score.  ^1^OH - Orthostatic Hypotension  Odds ratios (ORs) and 95% confidence intervals (CIs) for the association between OH and falls.  **Bold values** refer to a significant p-value (<0.05). | | | | | | | |

**Additional File 6: Logistic regression between sOH^1^ and falls, (n=404, 137 falls) – with covariates OR and CI 95%**

|  | Participants with falls/n | Model^a^ | Model^b^ | Recovery Categories | Participants with falls/n | Model^a^ | Model^b^ |
| --- | --- | --- | --- | --- | --- | --- | --- |
| No sOH | 104/329 | Ref. | Ref | No sOH | 104/329 | Ref. | Ref |
| sOH | 33/75 | **2.00**  **[1.15, 3.47]** | **2.05**  **[1.17, 3.61]** | sOH with Early Recovery | 16/48 | 1.20  [0.60, 2.38] | 1.23  [0.62, 2.45] |
|  |  |  |  | sOH with Delayed Recovery | 12/19 | **4.73**  **[1.71, 13.10]** | **4.97**  **[1.78, 13.87]** |
|  |  |  |  | sOH with No Recovery | 5/8 | **5.06**  **[1.42, 22.27]** | **5.97**  **[1.30, 27.38]** |
| Sex |  | 1.08  [0.58, 2.00] | 1.03  [0.54, 1.97] |  |  | 1.01  [0.59, 2.06] | 1.07  [0.55, 2.06] |
| Age |  | 0.96  [0.91, 1.01] | 0.96  [0.91, 1.01] |  |  | 0.96  [0.91, 1.02] | 0.96  [0.91, 1.02] |
| Diabetes |  | 1.03  [0.54, 1.99] | 1.09  [0.56, 2.11] |  |  | 1.04  [0.54, 2.02] | 1.09  [0.56, 2.14] |
| IADL |  | **1.10**  **[1.01, 1.20]** | 1.09  [0.99, 1.20] |  |  | **1.12**  **[1.02, 1.22]** | **1.10**  **[1.00, 1.22]** |
| Hand Grip Strength |  | 0.99  [0.96, 1.03] | 0.99  [0.96, 1.03] |  |  | 1.00  [0.97, 1.03] | 1.00  [0.96, 1.03] |
| FRIDs |  | 0.99  [0.82, 1.19] | 1.00  [0.82, 1.20] |  |  | 0.96  [0.80, 1.16] | 0.97  [0.80, 1.17] |
| HADS-A score |  | 1.42  [0.76, 2.65] | 1.43  [0.75, 2.73] |  |  | 1.52  [0.81, 2.84] | 1.55  [0.80, 2.98] |
| sFES-I score |  | 1.05  [0.96, 1.15] | 1.04  [0.94, 1.15] |  |  | 1.04  [0.95, 1.14] | 1.03  [0.93, 1.14] |
| Falls in the prior 12 months |  | **2.18**  **[1.38, 3.43]** | **2.25**  **[1.41, 3.59]** |  |  | **2.29**  **[1.44, 3.62]** | **2.39**  **[1.49, 3.84]** |
| Baseline SBP |  |  | 1.00  [0.98, 1.01] |  |  |  | 0.99  [ 0.98, 1.01] |
| ^a^Adjusted for age, sex, diabetes, instrumental Activities of Daily Living (iADL) score, hand grip strength, Fall Risk-Increasing Drugs (FRIDs), falls in the prior 12 months, Falls Efficacy Scale-International (FES-I) score, Hospital Anxiety and Depression Scale (HADS)-anxiety score.  ^1^sOH- systolic Orthostatic Hypotension  Odds ratios (ORs) and 95% confidence intervals (CIs) for the association between sOH and falls.  **Bold values** refer to a significant p-value (<0.05). | | | | | | | |

**Additional File 7: Logistic regression between OHTN^1^ and falls (n=357, 115 falls) – with covariates OR and CI 95%**

|  | Participants with falls/n | Model^a^ | Model^b^ | Recovery Categories | Participants with falls/n | Model^a^ | Model^b^ |
| --- | --- | --- | --- | --- | --- | --- | --- |
| No OHTN | 93/279 | Ref. | Ref | No OHTN | 93/279 | Ref. | Ref |
| OHTN | 22/78 | 1.05  [0.58, 1.92] | 1.09  [0.59, 1.99] | OHTN with Early Recovery | 6/29 | 0.62  [0.23, 1.67] | 0.64  [0.24, 1.75] |
|  |  |  |  | OHTN with Delayed Recovery | 9/18 | 2.49  [0.88, 7.10] | 2.65  [0.93, 7.59] |
|  |  |  |  | OHTN with No Recovery | 7/31 | 0.95  [0.38, 2.39] | 0.95  [0.37, 2.41] |
| Sex |  | 1.35  [0.80, 2.29] | 1.33  [ 0.77, 2.31] |  |  | 1.27  [0.75, 2.16] | 1.25  [ 0.72, 2.18] |
| Age |  | 0.95  [0.89, 1.00] | 0.94  [0.89, 1.00] |  |  | 0.94  [0.89, 1.00] | 0.94  [0.88, 1.00] |
| Diabetes |  | 1.25  [0.54, 2.89] | 1.34  [0.57, 3.13] |  |  | 1.26  [0.54, 2.94] | 1.36  [ 0.57, 3.21] |
| IADL |  | **1.15**  **[1.04, 1.28]** | **1.16**  **[1.02, 1.32]** |  |  | **1.15**  **[1.03, 1.28]** | **1.16**  **[1.02, 1.31]** |
| Falls in the prior 12 months |  | **3.49**  **[2.09, 5.84]** | **3.69**  **[ 2.17, 6.26]** |  |  | **3.51**  **[2.08, 5.90]** | **3.70**  **[2.16, 6.33]** |
| Hemoglobin |  | 0.89  [0.73, 1.09] | 0.91  [0.74, 1.12] |  |  | 0.90  [0.73, 1.10] | 0.92  [0.74, 1.13] |
| Diuretic Treatment |  | 1.13  [0.67, 1.93] | 1.29  [0.74, 2.24] |  |  | 1.11  [0.65, 1.90] | 1.26  [0.72, 2.21] |
| sFES-I score |  | 1.05  [0.94, 1.17] | 1.04  [0.92, 1.18] |  |  | 1.05  [0.94, 1.17] | 1.05  [0.92, 1.19] |
| HADS-A score |  | 1.45  [0.72, 2.94] | 1.50  [0.72, 3.12] |  |  | 1.50  [0.74, 3.06] | 1.56  [0.74, 3.28] |
| SBP Baseline |  |  | 0.99  [0.98, 1.01] |  |  |  | 0.99  [0.98, 1.01] |
| ^a^Adjusted for age, sex, diabetes, instrumental Acitvities of Daily Living (iADL), falls in the previous 12 months, hemoglobin, use of diuretics, Falls Efficacy Scale-International (FES-I) and Hospital Anxiety and Depression Scale (HADS)-anxiety-score  ^1^OHTN- Orthostatic Hypertension  Odds ratios (ORs) and 95% confidence intervals (CIs) for the association between OHTN and falls.  **Bold values** refer to a significant p-value (<0.05). | | | | | | | |

**Additional File 8: Logistic regression between sOHTN^1^ and falls (n=408, 111 falls) – with covariates OR and CI 95%**

|  | Participants with falls/n | Model^a^ | Model^b^ | Recovery Categories | Participants with falls/n | Model^a^ | Model^b^ |
| --- | --- | --- | --- | --- | --- | --- | --- |
| No sOHTN | 111/363 | Ref. | Ref | No sOHTN | 111/363 | Ref. | Ref |
| sOHTN | 11/45 | 1.02  [0.48, 2.16] | 1.01  [0.47, 2.16] | sOHTN with Early Recovery | 3/18 | 0.66  [0.18, 2.44] | 0.64  [0.17, 2.39] |
|  |  |  |  | sOHTN with Delayed Recovery | 5/14 | 1.51  [0.46, 5.03] | 1.55  [0.47, 5.16] |
|  |  |  |  | sOHTN with No Recovery | 3/13 | 1.05  [0.27, 4.05] | 1.03  [0.27, 4.00] |
| Sex |  | 1.35  [0.82, 2.22] | 1.34  [0.80, 2.25] |  |  | 1.34  [0.81, 2.21] | 1.33  [0.79, 2.23] |
| Age |  | 0.95  [0.90, 1.00] | 0.95  [0.89, 1.00] |  |  | 0.95  [0.90, 1.00] | 0.94  [0.89, 1.00] |
| Diabetes |  | 1.02  [0.48, 2.16] | 1.28  [0.60, 2.75] |  |  | 1.21  [0.57, 2.57] | 1.26  [0.59, 2.70] |
| IADL |  | **1.16**  **[1.05, 1.27]** | **1.16**  **[1.03, 1.29]** |  |  | **1.15**  **[1.04, 1.27]** | **1.15**  **[1.03, 1.29]** |
| Falls in the prior 12 months |  | **3.18**  **[1.96, 5.16]** | **3.30**  **[2.01, 5.43]** |  |  | **3.16**  **[1.94, 5.15]** | **3.28**  **[1.99, 5.41]** |
| Hemoglobin |  | 0.86  [0.71, 1.04] | 0.88  [0.72, 1.07] |  |  | 0.86  [0.71, 1.05] | 0.88  [0.72, 1.08] |
| Diuretic Treatment |  | 1.15  [0.70, 1.89] | 1.27  [0.76, 2.13] |  |  | 1.16  [0.71, 1.92] | 1.29  [0.77, 2.17] |
| sFES-I score |  | 1.05  [0.94, 1.16] | 1.04  [0.93, 1.17] |  |  | 1.05  [0.95, 1.17] | 1.05  [0.94, 1.18] |
| HADS-A score |  | 1.34  [0.68, 2.64] | 1.38  [0.68, 2.78] |  |  | 1.35  [0.69, 2.66] | 1.39  [0.69, 2.80] |
| SBP Baseline |  |  | 1.00  [0.98, 1.01] |  |  |  | 1.00  [0.98, 1.01] |
| ^a^Adjusted for age, sex, diabetes, instrumental Acitvities of Daily Living (iADL), falls in the previous 12 months, hemoglobin, use of diuretics, Falls Efficacy Scale-International (FES-I) and Hospital Anxiety and Depression Scale (HADS)-anxiety-score  ^1^sOHTN – systolic Orthostatic Hypertension  Odds ratios (ORs) and 95% confidence intervals (CIs) for the association between sOHTN and falls.  **Bold values** refer to a significant p-value (<0.05). | | | | | | | |

**Missings in Covariates:**

| **Additional File 9 for OH vs no OH** | | | | | | | | | | | | | | | | | | | | | | | |
| --- | --- | --- | --- | --- | --- | --- | --- | --- | --- | --- | --- | --- | --- | --- | --- | --- | --- | --- | --- | --- | --- | --- | --- |
| Data entries | | | | Sex | | Age | | Diabetes | | IADL | | Hand Grip Strength | | FRIDS medications | | HADS | | FES-I | | Falls in the prior 12 months | | |  |
| N | | Valid | | 430 | | 430 | | 427 | | 428 | | 428 | | 394 | | 430 | | 423 | | 426 | | |  |
|  |  | Missing | | 0 | | 0 | | 3 | | 2 | | 2 | | 36 | | 0 | | 7 | | 4 | | |  |
| **Additional File 10 for sOH vs no sOH** | | | | | | | | | | | | | | | | | | | | | |  |  |
| Data entries | | | Sex | | Age | | Diabetes | | IADL | | Hand Grip Strength | | FRIDS medications | | HADS | | FES-I | | Falls in the prior 12 months | |  |  |  |
| N | Valid | | 463 | | 463 | | 460 | | 461 | | 461 | | 420 | | 463 | | 456 | | 459 | |  |  |  |
|  | Missing | | 0 | | 0 | | 3 | | 2 | | 2 | | 43 | | 0 | | 7 | | 4 | |  |  |  |

| **Additional File 11 for OHTN vs no OHTN** | | | | | | | | | | | |
| --- | --- | --- | --- | --- | --- | --- | --- | --- | --- | --- | --- |
| Data entries | | Sex | Age | Falls | Diabetes | IADL | Falls in the prior 12 months | Hemoglobin | Diuretic MEDs | sFES-I | HADS-A >8p |
| N | Valid | 372 | 371 | 372 | 369 | 372 | 368 | 369 | 372 | 366 | 372 |
|  | Missing | 0 | 1 | 0 | 3 | 0 | 4 | 3 | 0 | 6 | 0 |

| **Additional File S12 for sOHTN vs no sOHTN** | | | | | | | | | | | | |
| --- | --- | --- | --- | --- | --- | --- | --- | --- | --- | --- | --- | --- |
| Data entries | | Sex | Age | Falls | Diabetes | IADL | Falls in the prior 12 months | Hemoglobin | Diuretic MEDs | sFES-I | HADS-A > 8p |  |
| N | Valid | 425 | 424 | 425 | 422 | 424 | 421 | 422 | 425 | 418 | 425 |  |
|  | Missing | 0 | 1 | 0 | 3 | 1 | 4 | 3 | 0 | 7 | 0 |  |

**Additional Files 13 -16 Recovery Categories and Covariates – Descriptive Analysis**

| **Additional File 13- OH recovery groups + Reference 2 (N=430)** | | | | |
| --- | --- | --- | --- | --- |
| **Covariates** | **Reference Group N=293 (68,1%)** | **Early Recovery N=103 (24.0%)** | **Delayed Recovery N=24 (5.6%)** | **No Recovery n=10 (2.3%)** |
| **Female Sex, n (%)** | 135 (46.1) | 33 (32.0) | 8 (33.3) | 4 (40.0) |
| **Age, median (Q1, Q3)** | 79 (76, 84) | 78 (76, 82) | 79 (76, 82) | 82 (77, 83) |
| **Baseline SBP, median (Q1, Q3)** | 128 (116, 138) | 128 (118, 138) | 133 (126.3, 146) | 142 (130.5, 154.3) |
| **Baseline DBP, median (Q1, Q3)** | 73 (69, 78) | 73 (69, 78) | 75.5 (69.5, 79.8) | 75 (70.3, 82.3) |
| **Diabetes, n (%)** | 33 (11.4) | 18 (17.5) | 8 (33.3) | 3 (30.0) |
| **IADl (sum), median (Q1, Q3)** | 1 (0, 3) | 1 (0, 2) | 1 (0, 3) | 2 (1, 3) |
| **Falls in the prior 12 months, n (%)** | 88 (30.4) | 29 (28.2) | 7 (29.2) | 0 |
| **Hand Grip Strength, median (Q1, Q3)** |  |  |  |  |
| **Men** | 32.5 (26.8, 38.0) | 38.0 (32.8, 41.0) | 30.5 (22.0, 37.5) | 27.5 (27.0, 38.0) |
| **Women** | 18.0 (13.5, 22.0) | 20.0 (17.0, 24.0) | 20.0 (12.8, 32.5) | 23.5 (20.3, 27.5) |
| **FRIDS (sum), median (Q1, Q3)** | 2 (1, 3) | 2 (1, 3) | 3 (1, 3) | 3 (1, 3) |
| **sFES-I score median (Q1, Q3)** | 9 (7, 10) | 8 (7, 10) | 8 (7, 10) | 8 (8, 12) |
| **HADS-A <8 points, n (%)** | 45 (15.4) | 9 (8.7) | 2 (8.3) | 0 |

| **Additional File 14 - sOH Recovery Groups + Reference 1 (N=463)** | | | | |
| --- | --- | --- | --- | --- |
| **Covariates** | **Reference Group N=379 (81.9%)** | **Early Recovery**  **N=56 (12.1%)** | **Delayed Recovery**  **N=20 (4.3%)** | **No Recovery**  **N=8 (1.7%)** |
| **Female Sex, n (%)** | 170 (44.9) | 19 (33.9) | 8 (40.0) | 4 (50.0%) |
| **Age, median (Q1, Q3)** | 79 (76, 83) | 79 (77, 82) | 79 (76, 82) | 80 (76, 82) |
| **Baseline SBP, median (Q1, Q3)** | 128 (116, 138.5) | 130.5 (121.3, 145) | 133 (126.3, 146) | 148.5 (133, 158.8) |
| **Baseline DBP, median (Q1, Q3)** | 73 (69, 78) | 74 (69.3, 78.8) | 74 (69.5, 78.8) | 77.5 (68.8, 84.8) |
| **Diabetes, n (%)** | 43 (11.4) | 13 (23.2) | 5 (25.0) | 2 (25.0) |
| **IADl (sum), median (Q1, Q3)** | 1 (0, 3) | 1 (0, 3) | 1 (0, 3) | 2 (1.3, 3) |
| **Falls in the prior 12 months, n (%)** | 110 (29.3) | 17 (30.4) | 6 (30.0%) | 0 |
| **Hand Grip Strength, median (Q1, Q3)** |  |  |  |  |
| **Men** | 34.0 (28.0, 39.0) | 36.0 (33.0, 40.0) | 30.5 (22.0, 38.0) | 27.5 (22.8, 36.0) |
| **Women** | 18.5 (14.0, 22.8) | 22.0 (18.0, 25.0) | 20.0 (12.8, 32.5) | 23.5 (20.3, 27.5) |
| **FRIDS (sum), median (Q1, Q3)** | 2 (1, 3) | 2 (1, 3) | 3 (1, 3) | 2 (1, 3) |
| **sFES-I score, median (Q1, Q3)** | 8 (7, 10) | 8 (7, 10) | 8 (7, 10) | 9 (8, 13) |
| **HADS-A <8 points, n (%)** | 53 (14.0) | 6 (10.7) | 1 (5.0) | 0 |

| **Additional File 15 - OHTN Recovery Groups + Reference 2 (N= 372)** | | | | |
| --- | --- | --- | --- | --- |
| **Covariates** | **Reference Group N=293 (78.8%)** | **Early Recovery N=29 (7.8%)** | **Delayed Recovery N=18 (4.8%)** | **No Recovery n=32 (8.6%)** |
| **Female Sex, n (%)** | 135 (46.1) | 14 (48.3) | 14 (77.89 | 16 (50.0) |
| **Age, median (Q1, Q3)** | 79 (76, 84) | 78 (76. 83) | 80 (77, 85) | 81 (75, 84) |
| **Baseline SBP, median (Q1, Q3)** | 128 (116, 138) | 126 (115.5, 149) | 127.5 (121.5, 147.3) | 123 (116.3, 134.5) |
| **Baseline DBP, median (Q1, Q3)** | 73 (69, 78) | 75 (72.5, 79) | 73.5 (68.8, 80.3) | 71.5 (66, 74.5) |
| **Diabetes, n (%)** | 33 (11.4) | 0 | 0 | 4 (12.5) |
| **IADl (sum), median (Q1, Q3)** | 1 (0, 3) | 1 (0, 1) | 0 (0, 3.5) | 1 (0, 2) |
| **Falls in the prior 12 months, n (%)** | 88 (30.4) | 9 (31.0) | 5 (27.8) | 4 (12.5) |
| **Hand Grip Strength, median (Q1, Q3)** |  |  |  |  |
| **Men** | 32.5 (26.8, 38.0) | 32.0 (27.5, 36.0) | 19.0 (15.0, 26.0) | 34.0 (28.5, 39.5) |
| **Women** | 18.0 (13.5, 22.0) | 19.0 (13.5, 20.3) | 23.0 (14.0, 29.3) | 18.0 (12.3, 23.8) |
| **Hemoglobin (g/dL), median (Q1, Q3)** | 13.7 (12.8, 14.6) | 13.7 (13.1, 14.9) | 13.3 (12.6, 14.0) | 14.0 (13.5, 14.7) |
| **Diuretic Medication, n (%)** | 112 (38.2) | 7 (24.1) | 5 (27.8) | 4 (12.5) |
| **sFES-I score, median (Q1, Q3)** | 9 (7, 10) | 8 (7, 11) | 8 (7, 11, ) | 8 (7, 10) |
| **HADS-A <8 points, n (%)** | 45 (15.4) | 4 (13.8) | 1 (5.6) | 2 (6.3) |

| **Additional File 16 - sOHTN Recovery Groups + Reference 1 (N= 425)** | | | | |
| --- | --- | --- | --- | --- |
| **Covariates** | **Reference Group N=379 (89.2%)** | **Early Recovery N=18 (4.2%)** | **Delayed Recovery N=14 ( 3.3%)** | **No Recovery n=14 (3.3%)** |
| **Female Sex, n (%)** | 170 (44.9) | 8 (44.4) | 10 (71.4) | 5 (35.7) |
| **Age, median (Q1, Q3)** | 79 (76, 83) | 79 (76, 81) | 81.5 (77.8, 86) | 81 (74, 85) |
| **Baseline SBP, median (Q1, Q3)** | 128 (116, 138.5) | 119.5 (114, 127.3) | 122.5 (119.5, 135) | 123 (111, 135) |
| **Baseline DBP, median (Q1, Q3)** | 73 (69, 78) | 73 (69, 76.3) | 73.5 (67.3, 79.3) | 72 (65.8, 77.3) |
| **Diabetes, n (%)** | 43 (11.4) | 0 | 1 (7.1) | 2 (14.3) |
| **IADl (sum), median (Q1, Q3)** | 1 (0, 3) | 0.5 (0, 1.3) | 1 (0, 4.3) | 2 (0, 2) |
| **Falls in the prior 12 months, n (%)** | 110 (29.3) | 4 (22.2) | 4 (28.6) | 1 (7.1) |
| **Hand Grip Strength, median (Q1, Q3)** |  |  |  |  |
| **Men** | 34.0 (28.0, 39.0) | 32.0 (26.0, 43.5) | 23.5 (20.5, 34.8) | 34.0 (29.0, 39.0) |
| **Women** | 18.5 (14.0, 22.8) | 17.0 (10.5, 20.0) | 22.0 (13.0, 25.0) | 15.0 (13.5, 28.0) |
| **Hemoglobin (g/dL), median (Q1, Q3)** | 13.7 (12.9, 14.6) | 14.2 (13.6, 15.7) | 13.4 (12.6, 14.3) | 14.0 (13.7, 14.7) |
| **Diuretic Medication, n (%)** | 142 (37.5) | 4 (22.2) | 2 (14.3) | 3 (21.4) |
| **sFES-I score, median (Q1, Q3)** | 8 (7, 10) | 8.5 (7, 10) | 7.5 (7, 9.5) | 9 (7, 11) |
| **HADS-A <8 points, n (%)** | 53 (14.0) | 1 (5.6) | 0 | 2 (14.3) |
